# Molecular Targets of Aptamers in Gastrointestinal Cancers: Cancer Detection, Therapeutic Applications, and Associated Mechanisms

**DOI:** 10.7150/jca.85260

**Published:** 2023-08-06

**Authors:** Khang Wen Goh, Annatasha Stephen, Yuan Seng Wu, Maw Shin Sim, Kalaivani Batumalaie, Subash C.B. Gopinath, Rhanye Mac Guad, Ashok Kumar, Mahendran Sekar, Vetriselvan Subramaniyan, Neeraj Kumar Fuloria, Shivkanya Fuloria, Appalaraju Velaga, Md. Moklesur Rahman Sarker

**Affiliations:** 1 Faculty of Data Science and Information Technology, INTI International University, 71800 Nilai, Malaysia.; 2 Department of Pharmaceutical Life Sciences, Faculty of Pharmacy, University of Malaya, Kuala Lumpur 50603, Malaysia.; 3 Centre for Virus and Vaccine Research, School of Medical and Life Sciences, Sunway University, Selangor 47500, Malaysia.; 4 Department of Biological Sciences, School of Medical and Life Sciences, Sunway University, Selangor 47500, Malaysia.; 5 Department of Pharmaceutical Life Sciences, Faculty of Pharmacy, University of Malaya, Kuala Lumpur 50603, Malaysia.; 6 Department of Biomedical Sciences, Faculty of Health Sciences, Asia Metropolitan University, 81750 Johor Bahru, Malaysia.; 7 Faculty of Chemical Engineering & Technology, Arau 02600, Institute of Nano Electronic Engineering, Kangar 01000, Micro System Technology, Centre of Excellence, Arau 02600, Pauh Campus, Universiti Malaysia Perlis (UniMAP), Perlis, Malaysia.; 8 Institute of Nano Electronic Engineering, Universiti Malaysia Perlis (UniMAP), 01000 Kangar, Perlis, Malaysia.; 9 Micro System Technology, Centre of Excellence (CoE), Universiti Malaysia Perlis (UniMAP), Pauh Campus, 02600 Arau, Perlis, Malaysia.; 10 Department of Computer Science and Engineering, Faculty of Science and Information Technology, Daffodil International University, Daffodil Smart City, Birulia, Savar, Dhaka 1216, Bangladesh.; 11 Department of Biomedical Science and Therapeutics, Faculty of Medicine and Health Science, Universiti Malaysia Sabah, Kota Kinabalu, Sabah, Malaysia.; 12 Department of Internal Medicine, University of Kansas Medical Centre, Kansas City, Kansas 66103, United States.; 13 School of Pharmacy, Monash University Malaysia, Bandar Sunway, Subang Jaya 47500, Malaysia.; 14 Department of Pharmacology, Jeffrey Cheah School of Medicine and Health Sciences, MONASH University, Malaysia.; 15 Department of Pharmacology, School of Medicine, Faculty of Medicine, Bioscience and Nursing, MAHSA University, Selangor 42610, Malaysia.; 16 Centre of Excellence for Biomaterials Engineering & Faculty of Pharmacy, AIMST University, Bedong 08100, Malaysia.; 17 Center for Transdisciplinary Research, Department of Pharmacology, Saveetha Institute of Medical and Technical Sciences, Saveetha Dental College and Hospitals, Saveetha University, Chennai 600077, India.; 18 Faculty of Pharmacy, AIMST University, Semeling, Bedong 08100, Malaysia.; 19 Department of Medicinal Chemistry, Faculty of Pharmacy, MAHSA University, Selangor 42610, Malaysia.; 20 Department of Pharmacy, State University of Bangladesh, 77 Satmasjid Road, Dhanmondi, Dhaka 1205, Bangladesh.; 21 Health Med Science Research Network, 3/1, Block F, Lalmatia, Dhaka 1207, Bangladesh.

**Keywords:** Alimentary and digestive tract cancer, single-stranded oligonucleotides, therapeutic targets, cancer diagnosis, cancer prognosis, drug delivery

## Abstract

Gastrointestinal (GI) cancers are among the most common cancers that impact the global population, with high mortality and low survival rates after breast and lung cancers. Identifying useful molecular targets in GI cancers are crucial for improving diagnosis, prognosis, and treatment outcomes, however, limited by poor targeting and drug delivery system. Aptamers are often utilized in the field of biomarkers identification, targeting, and as a drug/inhibitor delivery cargo. Their natural and chemically modifiable binding capability, high affinity, and specificity are favored over antibodies and potential early diagnostic imaging and drug delivery applications. Studies have demonstrated the use of different aptamers as drug delivery agents and early molecular diagnostic and detection probes for treating cancers. This review aims to first describe aptamers' generation, characteristics, and classifications, also providing insights into their recent applications in the diagnosis and medical imaging, prognosis, and anticancer drug delivery system of GI cancers. Besides, it mainly discussed the relevant molecular targets and associated molecular mechanisms involved, as well as their applications for potential treatments for GI cancers. In addition, the current applications of aptamers in a clinical setting to treat GI cancers are deciphered. In conclusion, aptamers are multifunctional molecules that could be effectively used as an anticancer agent or drug delivery system for treating GI cancers and deserve further investigations for clinical applications.

## 1. Introduction

Gastrointestinal (GI) cancers can be broadly classified as esophageal, gastric or stomach, liver, colorectal, and pancreatic cancers. Globally, they contribute approximately 4.2 million new cases and 3.1 million deaths reported in 2020, accounting for approximately 22% of total cancer cases and 32% of total cancer-related deaths. Among these, colorectal cancer (CRC) is a principal malignant condition that contributes to approximately 1.1 million new cases, followed by 1,089,103 stomach/gastric cancer (GC), 905,677 liver cancer (LC), 604,100 esophageal cancer (EC), and 495,773 million cases for pancreatic cancer (PC) worldwide 1. Although they share similar risk factors differ in etiology and epidemiological profiles 2. It has been reported that the incidences of GI cancers are declining globally, but the treatment outcome of GI cancer patients is still unsatisfactory, primarily due to poor diagnosis.

Identifying biomarkers based on clinical features and gene analysis can significantly improve the diagnosis, prognosis, and cancer treatment outcome. Cancer biomarkers determine the significant molecular pathways associated with cancer recurrence and treatment efficiencies 3. One of the major factors causing poor diagnosis in the early stages of GI cancers is they remain asymptomatic until they progress to an advanced stage 4. Generally, biomarkers are characteristics that are objectively measured and evaluated during a disease progression or treatment, and they indicate a biological, pathological, or pharmacological response. Several biomarkers have been identified for GI cancers, including carbohydrate antigen CA72-4, CA12-5, BCA-225, human chorionic gonadotrophin (hCG), pepsinogen I/II, carcinoembryonic antigen (CEA), and CA19-9, with CEA and CA19-9 are the most widely used biomarkers in clinical diagnosis 5. CEA is commonly associated with digestive tract cancer and is an independent risk factor for predictive liver metastasis relapse. Given that its levels are generally elevated in advanced stages, thus it is not a highly recommended screening biomarker for early-stage detection 6. Nonetheless, its presence in the peritoneal fluid can also indicate GC relapse, such as a repetitive recurrence of cancer cells in patients **7**. On the other hand, CA19-9 is a glycolipid antigen used as a biomarker for CRC. It is a selective ligand for E-selectin found on endothelial cells 8 and has a 56% sensitivity for recurrence with 74% specificity. It has been reported that CA19-9-positive GI cancers have an antral location, prominent lymphatic, and venous invasion, with metastasis in lymph nodes, thus providing useful information on metastasis and recurrence. Intriguingly, the sensitivity of CA19-9 increases significantly to 87% when combined with CEA 9. Besides, CA72-4, CA 125, and alpha-fetoprotein (AFP) are other biomarkers commonly used in diagnosing GI cancers. The limitations of molecular targets are their sensitivity and specificity. Some characteristics of the abovementioned biomarkers are yet to be discovered and evaluated for their sensitivity and specificity as a tool for diagnosis and prognosis.

Several biomarkers have been identified and developed in preclinical studies fail to get proper validation in clinical practice. For instance, HER2, a proto-oncogene encoded by human epidermal growth factor receptor 2 (ERBB2), is a cell membrane tyrosine receptor kinase and is the only biomarker available in clinical practice. Although its predictive value is debatable, it has emerged as an important diagnostic biomarker. HER2 gene amplification rate has shown to be 32% higher in intestinal-type cancer versus diffuse-type cancer. Trastuzumab, which mediates HER2 signaling, is perhaps the first small molecule inhibitor approved for GI cancer treatment 10. Quantitative detection of conventional serum or tissue biomarkers is a challenge in GI cancer biomarker discovery because of their insufficient specificity and sensitivity, plus their detection threshold is low and cannot be detected in most cases in the early stages. Therefore, there is a pressing need to find a reliable GI cancer biomarker.

Aptamers are short single-stranded oligonucleotides (DNA, RNA, or modified nucleic acid sequences) or peptides that bind strongly to a specific target. They are generated by the cell SELEX (Systematic Evolution of Exponential enrichment) method with a range of detecting and therapeutic applications. Aptamers are chemical analogs of antibodies and confer several appreciable benefits over the antibodies or small ligands, owing to their high stability at a high temperature, strong affinity to their target, and desirable small size over antibodies. Unlike antibodies, they can be synthesized and modified chemically to specifically bind to molecular targets without causing toxicity and are non-immunogenic. They have greater scalability and desirable storage properties and no batch-to-batch variation as commonly seen with antibodies. Their tissue permeability, desirable pharmacokinetic profiles, and modifiability make them suitable for diagnostic and therapeutic applications over antibodies 11. It has been proven that aptamers can be used to target molecules or cancer biomarkers and determine key downstream molecular signaling pathways to halt. This claim shows that aptamers can ultimately lead to cancer cell death and increase the impact and efficiency of treating cancer instead of options such as radiation or chemotherapy. One of the major drawbacks of chemotherapy is the non-selective nature of anticancer drugs, which subsequently lowers their therapeutic efficacy. Given aptamers' unique and natural features, they can be very selective toward cancer cells and thus have multiple applications in cancer diagnostics, targeting, and therapeutics. Besides, they can be conjugated with drugs to reach cancer cells or with any molecules, compounds, nanoparticles (NPs), etc., to formulate a targeted drug delivery system, thus minimizing the toxicity and increasing the therapeutic efficacy of chemotherapy as compared to the individual entity. Due to their advantage over antibodies, aptamers can be very useful in cancer diagnostics as they can be used to detect circulating tumor cells and *in vivo* imaging. Detecting circulating tumor cells can be challenging due to their deficient presence requiring highly efficient capturing. The efficiency can be enhanced based on aptamer-based binding of circulating tumor antigen 12 by combining with radionuclides, fluorescent molecules, or another imaging molecule. Aptamers have become a vital tool in the imaging diagnosis of cancer cells. This review discusses the current comprehensive understanding of aptamers in GI cancer as tools for diagnosis, prognosis, and treatments, including their application in clinical settings, drug delivery systems, and identification of specific molecular targets or cancer biomarkers.

## 2. Generation Technologies and Different Types of Aptamers

### 2.1. Cell SELEX Process

**Figure 1:**
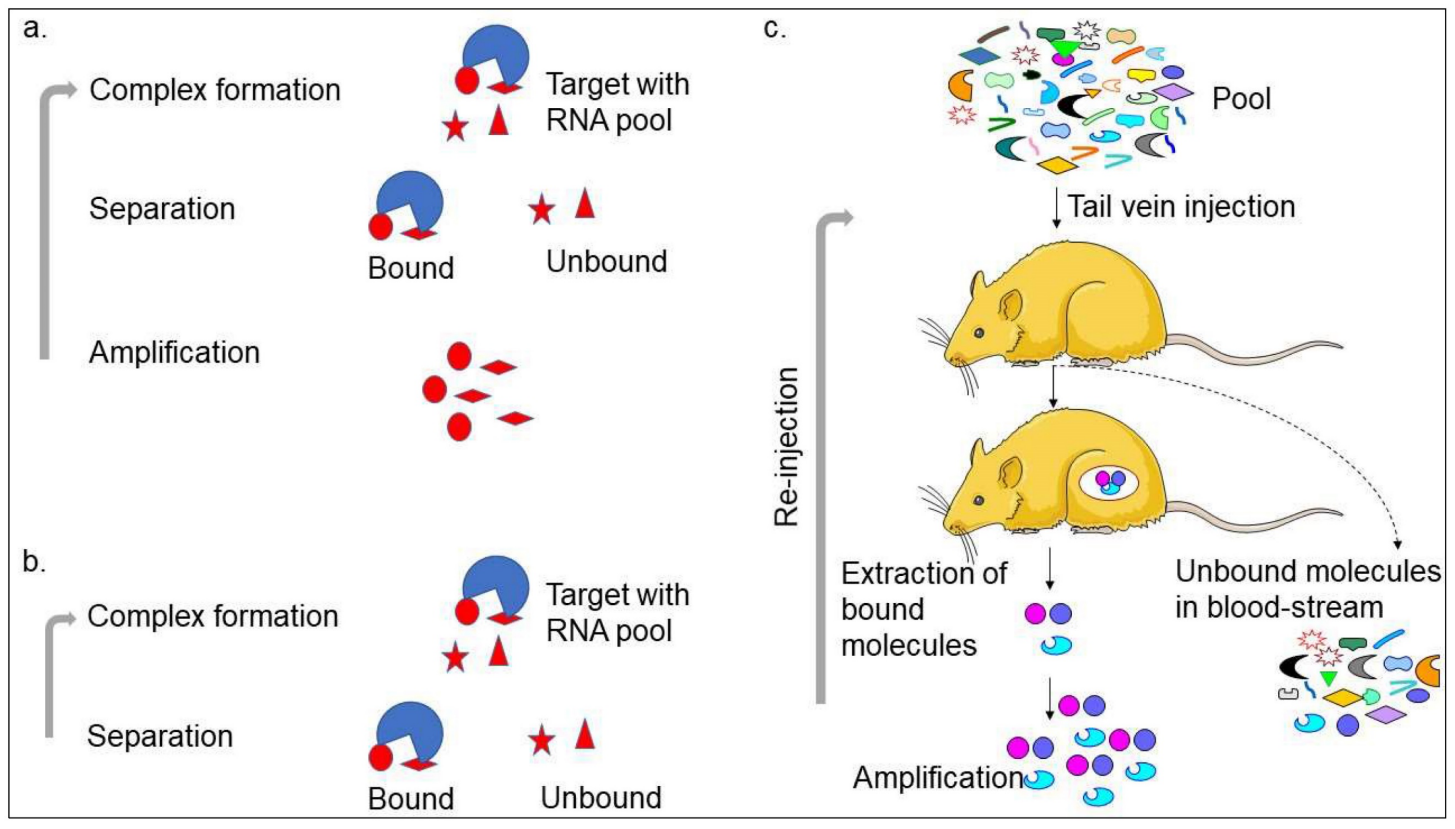
Strategies with aptamer production. a. Conventional SELEX; b. Non-SELEX; and c. *In vivo*-SELEX are shown with the steps involved. Other strategies generated for SELEX are following predominantly the conventional SELEX and *in vivo*-SELEX.

Aptamer or artificial antibody is the chemically synthesized oligonucleotide from the randomized library of molecules that is selected using the SELEX method involving binding, separation, and amplification (Figure 1a) 13. In the first step, the target molecule can bind with pool molecules, separating bound molecules from unbound molecules to the target molecule. The unbound molecules are removed through washing. The initial pool is selected carefully, with the randomized molecules varying from 25 bases (N25). The general concept is that the pool molecules with higher numbers can make more versatile structures and yield high-affinity aptamers at possible levels. However, a wide range of aptamers can be selected using different pools 13-15. The bound molecules are separated from the unbound molecules by several partition methods, such as titer plate, filter, magnetic bead, column matrix, etc. After eluting the bound molecules from the target, polymerase chain reaction (PCR)-amplification is used to enrich the bound molecules and used for the next round of SELEX. Five to 12 rounds are required to get the specific aptamer in most cases. With the cell SELEX selection, two research teams successfully generated the first aptamers 16, 17. After this, various aptamers were generated against a wide range of targets, such as toxins, organic molecules, peptides, proteins, carbohydrates, and antibiotics. Interestingly, it is also possible to generate aptamers against complex target structures and whole organisms 14, 15, 18, 19. With the continuous selection of different types of aptamers, the understanding of the SELEX procedure is progressed well in different ways. Ultimately, several SELEX methods have been established that can generate aptamers in a short time and use less reagents. These SELEX methods are complex target SELEX, Photo-SELEX, *in vivo* SELEX, SELEX with Beacons, Cell-SELEX, Genomic SELEX, Spigelmers, MonoLEX, and in silico SELEX. Recently, researchers have used a single-step method as non-SELEX in which the aptamer is selected without the amplification process (Figure 1a-c). The microfluidic device-based aptamer selection is one of the advanced levels of the selection process, which includes all the selection steps on one chip 20. The main benefit of the aptamer selection is that the SELEX process is carried out using *in vitro* and does not require used animals like antibody production. Besides, it is also possible to select aptamers against a wide range of non-immunogenic targets.

### 2.2. Types and Characteristics of Aptamers

Three major types of aptamers are DNA, RNA, and peptide aptamers, among which both DNA and RNA aptamers are widely used in many fields. Comparatively, DNA aptamer is more stable but less reactive than RNA aptamer, while RNA aptamer can make more secondary-dimensional structures than DNA aptamer. Additionally, it is easier to synthesize and stabilize RNA aptamer by simple chemical modification than DNA aptamer. Peptide aptamers are short amino acid sequences present within a scaffold protein. The short amino acids that are double-constrained within the protein are the target protein binding agent, and the protein aids in increasing the specificity and binding affinity 21.

DNA and RNA aptamers can create specific binding sites in their targets with adaptive conformational changes that alter the surface area of their targets. Contrarily, peptide aptamers create a smaller binding site that allows accurate target selection. DNA, RNA, and peptide aptamers exhibit different functional group variations, increasing the possibility of determining ideal targets 22. The comparison of highlighted properties between DNA, RNA, and peptide aptamers is presented in Table 1.

Aptamers are carefully selected after a certain round, and their binding affinity with the target is usually in the low-nanomolar to the low-femtomolar range. Moreover, aptamers are more specific to their targets; thus, they could discriminate among closely related molecules. This claim implies that they can distinguish the epitope on the target molecule, for instance, discriminate between the phosphorylated and dephosphorylated states. They can also differentiate the enantiomers of chiral molecules. Interestingly, because they are tiny, they can be delivered easier and can reach smaller areas in the biological system. Apart from that, aptamers have a versatile structure and can make various shapes, such as stems, loops, bulges, hairpins, pseudoknots, triplexes, and quadruplexes. Their secondary structures may fold into complex tertiary structures, permitting them to make complementary shapes wrap around or part of the target or fit snugly into clefts and gaps within the surface of larger targets 23. In most cases, aptamers bind with their targets in the loop region 14, 15. Because of the structural variation, they can bind with their targets through electrostatic interaction or hydrogen bonding, as well as possible interaction with aromatic compounds and nucleobases. Besides, aptamer binding to a target can occur in three different ways, including aptamer-induced targets, targets-induced aptamers, or both. With these versatile structures, aptamers are widely utilized in several fields, particularly biosensing due to their high affinity and selectivity.

### 2.3. Aptamer Targets Highly Expressed Molecules for Cancer Diagnosis and Molecular Imaging

**Figure 2:**
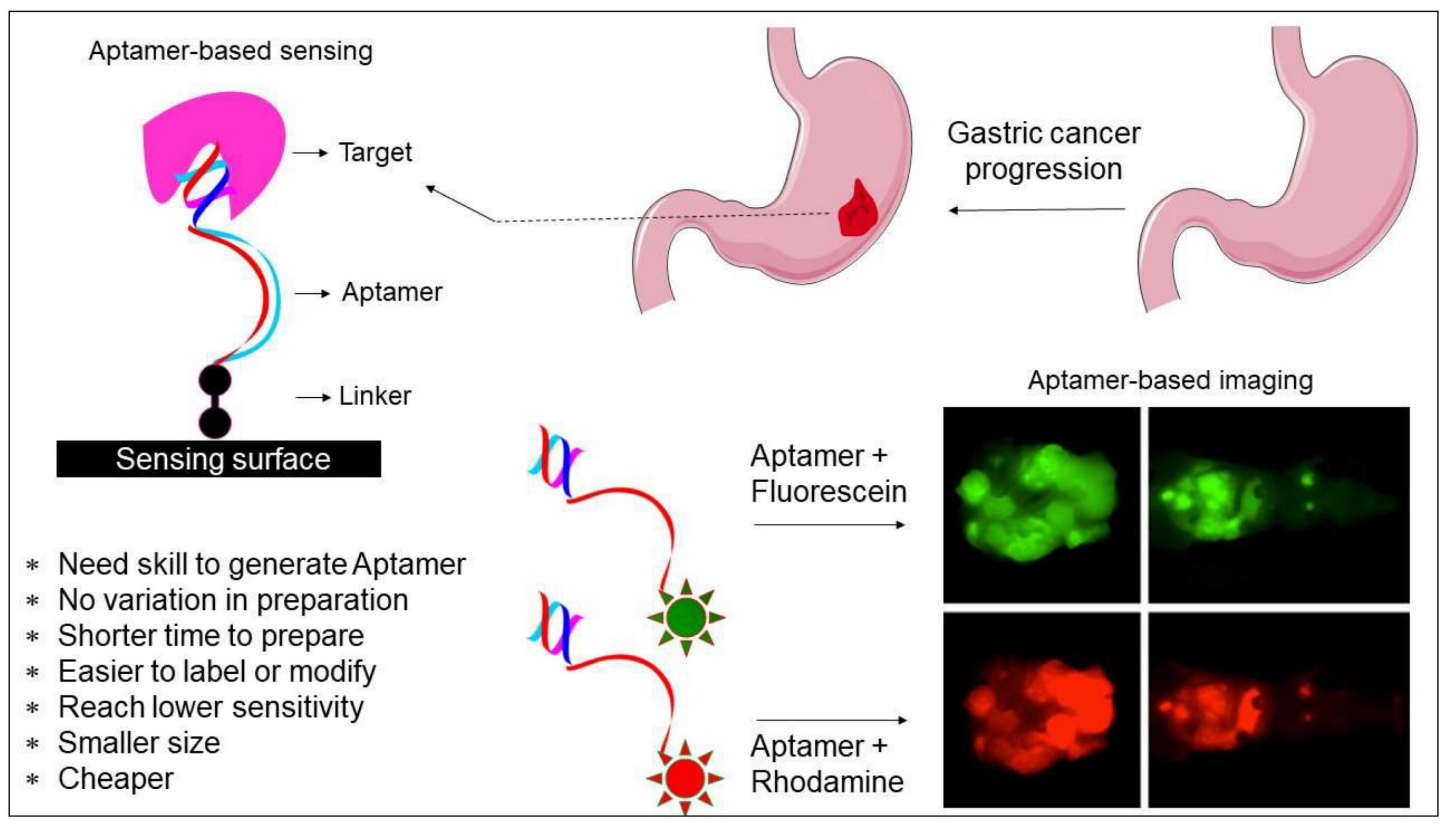
Potentials of aptamer in diagnosis. Both sensing and imaging-based identifications are shown. The advantages of aptamer and its suitability with different fluorescent compounds are displayed.

Aptamers are widely applied in many fields, such as biosensors, drug carriers, imaging, drug delivery, and medicine 24, 25. Given that aptamers have high affinity and selectivity with the target molecules, their application focuses more on biosensor development (Figure 2) 26, 27. It is essential to differentiate the closely related molecules to treat diseases and avoid spreading in medical diagnosis. Because only a few bases in the aptamer are involved in binding with the molecular targets; thus, they can differentiate from the closely related molecules. For instance, aptamers can differentiate the closely related strains within influenza virus types 15. Due to the high selectivity of aptamers, they can be used to detect the analyte molecules in crude samples. For instance, Lakshmipriya et al. 28 found that aptamers could detect factor IX protein from human serum and reached the limit of detection (LOD) to the lower picomolar range, acknowledging that the LOD with aptamers can be as low as picomolar to femtomolar for diagnosing and in medical imaging 29-31. Additionally, aptamers can also be arranged in the sandwich pattern using two aptamers with different binding sites.

Several studies have reported that aptamers can be useful tools in molecular imaging and diagnosis. Chen X et al. 32 proved that S3-2-3 aptamer was introduced to target esophageal squamous cell carcinoma (ESCC) KYSE150 cells for confocal tissue imaging, with fluorescent signals present and detected. This finding indicates that S3-2-3 aptamer can be used as a molecular imaging probe. Similarly, cy-apt 20 aptamer labeled with fluorescein isothiocyanate (FITC) successfully detected GC AGS cells with the emittance of strong fluorescence intensity 18, 33. Next, Xu J et al. 34 indicated that a few selected aptamer sequences (i.e., JHIT1, JHIT2, JHIT3, JHIT4, JHIT5, JHIT6, and JHIT7) could differentiate between normal and LC cells through the binding analysis method, implying that aptamers can also be used for cancer diagnosis. Wan J et al. 35 also proved that selected DNA aptamers (i.e., yl19, yl19a, and yl23) could recognize and bind to the membrane proteins as potential molecular targets to assist in identifying LC cells in QBC-939, a type of human cholangiocarcinoma cell line, thus contributing to cancer diagnosis. Rong Y et al. demonstrated that LY-1 aptamer bound to the overexpressed Cytokeratin 19 (CK19) and vimentin in metastatic LC cells, indicating cancer cells in pulmonary cancer tissues and local LC tissues. This finding shows that LY-1 aptamer can be used as a diagnostic tool and in molecular imaging 36. Moreover, the Apt-07S aptamer developed by Yu XX et al. recognized LC cells and differentiated them from normal cells by targeting Plk1 protein 37. These studies have proved that aptamer can be used as a molecular imaging tool in GC and LC cancer cells by labelling with fluorescence and used as a diagnostic probe through their binding specificity and selectivity of highly expressed molecules in cancer cells that are lowly expressed in normal cells.

A study by Dong et al. discovered a truncated aptamer, AP613-1, specifically bound to Glypican-3 (GPC3) protein in GPC3-positive hepatocellular carcinoma (HCC) cells. The modified AP613-1 are aptamers with bases at both 5' and 3' terminals that are replaced by phosphorothioates, and these aptamers showed a higher binding affinity than the unmodified AP613-1 aptamer. Phosphorothioate-modified AP613-1 binding to GPC3 showed potential in staining HCC cells for *in vivo* targeted imaging 38. Next, the ApC1 aptamer also showed potential in molecular imaging and cancer diagnosis. It specifically targeted colon Caco-2 cancer cells with CD133 high expression as compared to HEK293 normal cells and other cancer type cells and emitted fluorescence signals in confocal tissue imaging, showing its potential to be a diagnosis tool on top of being a molecular imaging tool 39. Collectively, truncated aptamer can improve the binding affinity of aptamer to highly expressed molecules like GPC3 in HCC, while ApC1 aptamer can be used as molecular imaging and diagnostic tools in colon cancer cells by targeting membrane-bound CD133 specifically.

Besides, targeted therapy and diagnostic tool for PC were developed using a single-stranded DNA (ssDNA) aptamer termed XQ-2d to target Cluster of Differentiation 71 (CD71), which was overexpressed in PC 40. Interestingly, XQ-2d exhibited a high binding affinity against PC cells. Wu et al. (2019) further discovered that CD71 silencing prevented XQ-2d from attaching to PC cells, indicating that XQ-2d targets membrane-bound CD71 instead of total CD71. Additionally, it was found that XQ-2d and transferrin (Tf) shared the same binding site on CD71 but not the anti-CD71 antibody, as evidenced in a competitive examination. The distance between the binding sites of XQ-2d and anti-CD71 antibody was measured using a surface energy transfer (SET) nano-ruler, and it was roughly 15 nm. The spatial organization of XQ-2d and Tf competitively binding to CD71 was resolved using molecular dynamics modeling 40. In another study, researchers tested the specificity of Cy3 coupling with P19/P1 aptamers to be employed as a diagnostic tool on archival human pancreatic duodenectomy tissue slices of pancreatic ductal adenocarcinoma (PDAC) patients. According to a scoring pattern from 72 patients, a favorable link was found between strong fluorescence signals in the high-mortality patient groups 41. Matrix metalloproteinase 14 (MMP14) is overexpressed in various cancers and is linked to a bad PC prognosis. As for MMP-14 positive pancreatic cancer cell lines, aptamer M17 was identified to specifically probe and recognize PC cells for molecular imaging and diagnosis. Aptamer M17 demonstrated its capability to target MMP-14 and contributed to *in vivo* and *in vitro* tumor imaging 42. It has been reported that modified DNA aptamers with amino acid-like side chains or drug-like ligands can provide unique benefits and improve specificity 43. For instance, Thy-1 membrane glycoprotein (THY1 or CD90) has been discovered as an overexpressed neovasculature biomarker in PDAC. Thus, Wang et al. designed and tested modified DNA X-aptamers targeting THY1. After library screening and affinity binding evaluation, three high-affinity THY1 X-aptamers (i.e., XA-B217, XA-B216, and XA-A9) were chosen. Using the THY1 antibody as a reference, these three X-aptamers displayed good binding affinity and specificity to THY1 protein and THY1-expressing PDAC cells. In the context of biomarker discovery and therapeutic applications, the development of these X aptamers provides highly selective and non-immunogenic affinity ligands for THY1 binding; thus, they could be used to aid molecular imaging of PDAC that targets THY1 43. Thus, aptamers like XQ-2D, P19/P1, THY1 X can be used as molecular imaging and diagnostic tools in PC by binding to membrane-bound CD71, MMP-14, and THYI.

## 3. Aptamer-based Recognition of GI Cancer and Applications

### 3.1. Specific Detection for Different Cancer Types

Aptamers are mostly used in cancer diagnosis and have the advantage of recognizing cancer cells by binding to molecular targets. Therefore, they can determine the specific detection of GI cancer cells. For instance, Chen X et al. 32 utilized the cell-SELEX technology to successfully screen three different aptamers consisting of ssDNA sequences (i.e., S2, S3, and S8) with a strong affinity for ESCC KYSE150 cells. It was detected by flow cytometry assay that S2 aptamer showed specificity against KYSE150 cells, while S3 aptamer could significantly target KYSE150, KYSE30, KYSE140, EC109, TE-1, KYSE450, and KYSE70 cells. Next, the S8 aptamer showed a significant fluorescence signal in KYSE150, KYSE30, KYSE140, and KYSE70 cells, implying that these aptamers are ESCC-specific recognition molecules. Besides, Chen et al. used several truncating techniques to produce specific aptamers with higher binding affinity against ESCC cells by designing the secondary structure of nucleic acid systems using the Nucleic Acid Package (NUPACK) software. S2-1 and S2-2, S3-1 and S3-2, and S8-1, S8-2, and S8-3 were successfully obtained from S2, S3, and S8 aptamers, respectively 44. Following analysis by flow cytometry assay, S3-2-3 aptamer had the highest fluorescence intensity on truncated sequence optimization as evidenced by the flow cytometry analysis as compared to S3-2-1 and S3-2-2 aptamers, indicating that S3-2-3 aptamer is the best. Importantly, S3-2-3 aptamer also showed high binding affinity against KYSE30 cells, as indicated by the dissociation constant (Kd) in the nanomolar range (265.3±26.8 nM). An ultrashort S3-2-3 aptamer with 18 bases was then identified and labeled with Cy5 for testing its potential to be applied for ESCC tissue imaging, as this method is cheap, simple, and selective. The confocal imaging revealed that ESCC tissues displayed significant red fluorescence signals, implying an important role of this labeled aptamer in clinical application for diagnosis and target therapy of ESCC. Further analysis by proteinase K and sodium dodecyl sulfate-polyacrylamide gel electrophoresis (SDS-PAGE) showed that selected aptamers' targets were most likely membrane proteins, which may facilitate the discovery of biomarkers and targeted therapy of ESCC.

Alternatively, it was shown that epithelial cell adhesion molecule (EpCAM) protein could be targeted with aptamers. EpCAM is highly overexpressed in EC cell lines and has been reported to contribute to cancer metastasis to the adjacent organs and lead to poor prognosis 44. Given this, Liu et al designed an EpCAM-specific aptamer, known as SYL3C, to differentiate EC cells from normal cells instead of using antibodies. SYL3C aptamer successfully showed potential to be a reliable biomarker for EC through aptamer-based staining targeting EpCAM expression. Therefore, EpCAM protein staining with SYL3C did not only identify severe dysplastic EC cells but also differentiated lesions and benign cells, showing the diagnostic and prognostic values of SYL3C aptamer by targeting EpCAM 44.

Furthermore, Cao H-Y et al. 33 demonstrated the feasibility of using cy-apt 20 aptamer for detecting GC with high affinity and specificity, as indicated by fluorescence intensity emitted by bound GC AGS cells, which was seven times higher than LC HepG2 cells and SW620 CRC cells. This finding was confirmed in another study that the cy-apt 20 aptamers binding rate to AGS cells was >70%, while the binding affinity to non-GC cells was 30%, as measured by flow cytometry 18. The *in vitro* efficacy of cy-apt 20 aptamer was also supported by an *in vivo* study in which the fluorescence signals were detectable in GC in a dose and time-changing manner, as visualized by the IVIS Spectrum Imaging System. Fluorescence signals became evident 10 minutes after delivery of 1 nM cy5-labeled cy-apt 20 aptamers, followed by gradually reaching a peak at 120 minutes. In another study by Cao H-Y et al, they reported that the fluorescence intensity of AGS cells steadily increased after 40 minutes with increasing concentrations of FITC-cy-apt 20 aptamer and peaking at 400 nM 18. These findings show that targeted recognition can be established with a low dose of cy-apt 20 aptamer and can last sufficient for detection. Indirectly, it demonstrated that cy-apt 20 aptamer has acceptable specificity and sensitivity to target cells and is biostable *in vivo*.

According to Xu, J et al. 34, seven aptamer candidates (i.e., JHIT1, JHIT2, JHIT3, JHIT4, JHIT5, JHIT6, and JHIT7) showed apparent shifts in fluorescence intensity toward HepG2 HCC cells using flow cytometry, indicating that selected aptamers could distinguish HCC cells from normal liver cells. The Kd value further indicated that these aptamers were highly bound to HepG2 cells in the nanomolar range (64-349 nM). The absence of fluorescence shift after treating HepG2 cells with trypsin further confirmed that selected aptamers targeted membrane proteins. It was also noted that the seven mentioned aptamers were exclusively for MCF-7 breast cancer cells instead of MDA-MB-231 cells. Histologically, MCF-7 cells belong to the Luminal A subtype and have high expression of estrogen receptors (ER) and progesterone receptors (PR), which do not express in MDA-MB-231 cells, suggesting that these aptamers may be hormone-dependent. Besides, Apt-07S aptamer could specifically detect HCC HepG2 and SMMC-7721 cells with low Kd, 194.7±69.8 nM and 224.2±60.4 nM, respectively, as well as at a particularly specific binding capacity to two additional HCC cells, HCC-LM3 and Huh7 cells 37. According to the confocal imaging observation, Apt-07S aptamer only slightly bound to cells of other cancer types, such as MCF-7, H460, and SW480, as well as normal breast HBL-100 cells, indicating that Apt-07S aptamer has higher selectivity for HCC cells than normal cells. Furthermore, it was discovered that FAM-labeled Apt-07S aptamer was distributed both on the cell surface and in the cytoplasm of targeted cancer cells. This result implied that Apt-07S aptamer could precisely detect cancer cells and could be used as a possible carrier for transporting anticancer agents to cancer cells. Based on a cell growth inhibition rate study, Apt-07S aptamer was cytotoxic to HepG2 cells (>10 µM, p<0.05) and SMMC-7721 cells (>20 µM, p<0.05). Rong (2016) also discovered that Apt-07S aptamer integrated with ssDNA, Apt-07S-ASO-Plk1, was considerably more effective than antisense phosphorothioate oligodeoxynucleotide (ASODN)-polo-like kinase-1 (Plk1), at inhibiting HepG2 and SMMC-7721 cell growth (p<0.01), as well as less toxic to L02 normal liver cells 37.

Next, Wan, J et al. 35 showed that yl19 and yl23 aptamers exhibited a good binding capacity to QBC-939 cholangiocarcinoma cells, with equilibrium Kd values of 42.44±11.82 nM and 87.45±15.50 nM, respectively. Additionally, FAM-labeled yl19, yl19a, and yl23 aptamers demonstrated strong binding to QBC-939 cells while showing no significant recognition for HeLa cervical cancer cells; HepG2, Bel-7404, and SMMC-7721 HCC cells; CNE2 nasopharyngeal carcinoma cells, and A549 lung adenocarcinoma cells. When QBC-939 cells were treated with proteinase K, the fluorescence intensity on target cells containing aptamers yl19, yl19a, and yl23 was significantly reduced. This result indicated that the target molecules of these aptamers were most likely membrane proteins. The confocal imaging observation also showed that yl19 and yl23 aptamers could bind to the surface of target cells rather than control cells. Rong, Y et al 36 showed that LY-1 and LY-13 aptamers had high affinity against HCCLM9 HCC cells in which respective low Kds (167.3 ± 30.2 nM and 185.6 ± 28.3 nM) were measured. Fluorescence imaging and flow cytometry analyses further indicated that LY-1 aptamer demonstrated significant specific binding to HCCLM9 cells as compared to other HCC cell lines. It was also found that the targets of LY-1 aptamer were mainly membrane proteins on HCCLM9 cells after pretreatment with proteinase K. Besides, a study involving a xenograft model of HCC with pulmonary metastasis discovered that QD605-labeled LY-1 aptamer could detect both local LC tissues and metastatic lung tissues. The Western blotting analysis further confirmed the result that QD605-labeled LY-1 aptamer was capable of binding to a subpopulation of HCCLM9 cells, with a higher amount of CK19 and Vimentin presented, which are the protein markers associated with cancer migratory and invasiveness. Interestingly, QD605-labeled LY-1 aptamer treatment regulated metastatic HCC behaviors, as evidenced by a significant inhibition of cell migration (29.3 ± 3.06% of LY-1 treated cells vs 97.1 ± 2.11% of control cells; p<0.05) and cell invasion (53.8 ± 4.36% of LY-1 treated cells vs 96.83 ± 3.14% of control cells; p<0.05) in both non-coated and basal membrane coated (Matrig) HCC cells. Similarly, the xenograft mouse model transplanted with HCCLM9 cells also showed that LY-1 aptamer inhibited tumor growth after intraperitoneal injection (757.4±70.68 mm^3^) as compared to Phosphate-buffered saline, PBS, control (1962±139.8 mm^3^) and NK8-treated mice (1569±308.2 mm^3^). More importantly, the aptamers were non-toxic because mice did not show abnormal behavioral or body weight changes after treatment 36.

PC is usually not discovered until it has progressed to the point where surgery is no longer an option. In this regard, Ray et al. 45 designed an aptamer that could detect PC in the early stage. An *in vitro* positive or negative selection technique was established to identify RNA ligands (aptamers) that could detect structural variations between the secretomes of PC and non-cancerous cells to identify PC biomarkers. The molecular recognition technique discovered an aptamer (M9-5) that variably binds to conditioned media of malignant and non-cancerous human pancreatic cell lines. With great sensitivity and specificity, M9-5 aptamer could distinguish between the serum of PC patients and healthy individuals. Cyclophilin B (CypB) was identified as the target of M9-5 aptamer using biochemical purification procedures and mass spectrometric analysis. This molecular recognition-based approach discovered CypB as a serum biomarker while also developing a novel reagent to detect it in body fluids 45. In addition to cancer cells, Kim YJ et al. 46 also demonstrated that aptamers 1 and 146 could bind to cancer stem cells (CSCs) of the HPAC PC cells. They found that the binding affinity of aptamers 1 and 146 showed Kd values of 22.18 and 22.62 nM, respectively. Using Fluorescence-activated cell sorting (FACS) analysis, it was revealed that aptamer 1-positive cells had elevated levels of CSC-related genes (i.e., *CD133*, *CD24*, and *ALDH1*). On the other hand, aptamer 146-positive cells exhibited higher expression levels of CSC-associated genes, such as *CD133*, *CD24*, *IHH*, and *nanog*. Further analysis using a confocal microscope confirmed the colocalization of *CD44, CD24, ESA,* and *CD133* with both aptamers, indicating they bound to CSCs successfully.

**Figure 3:**
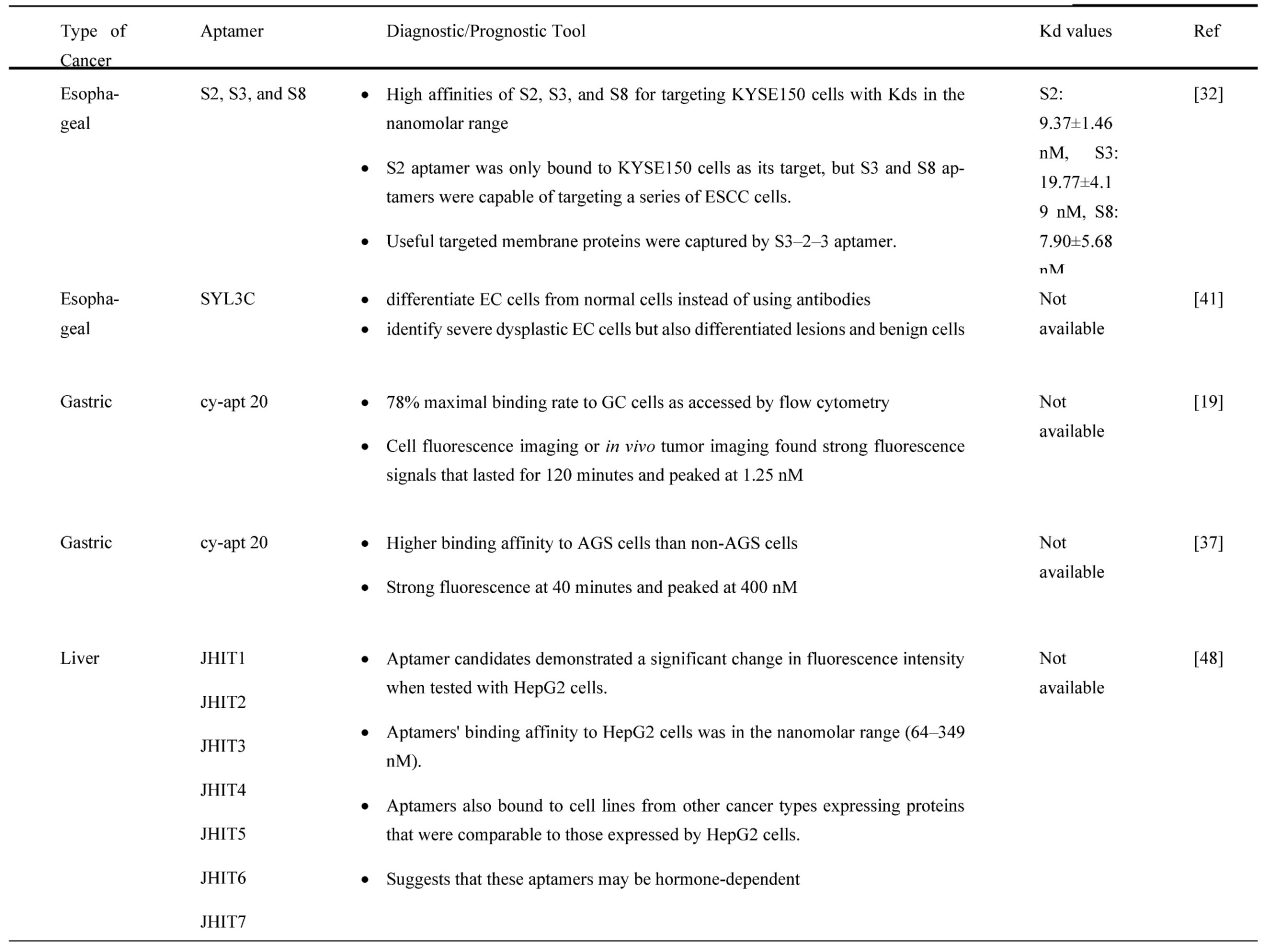

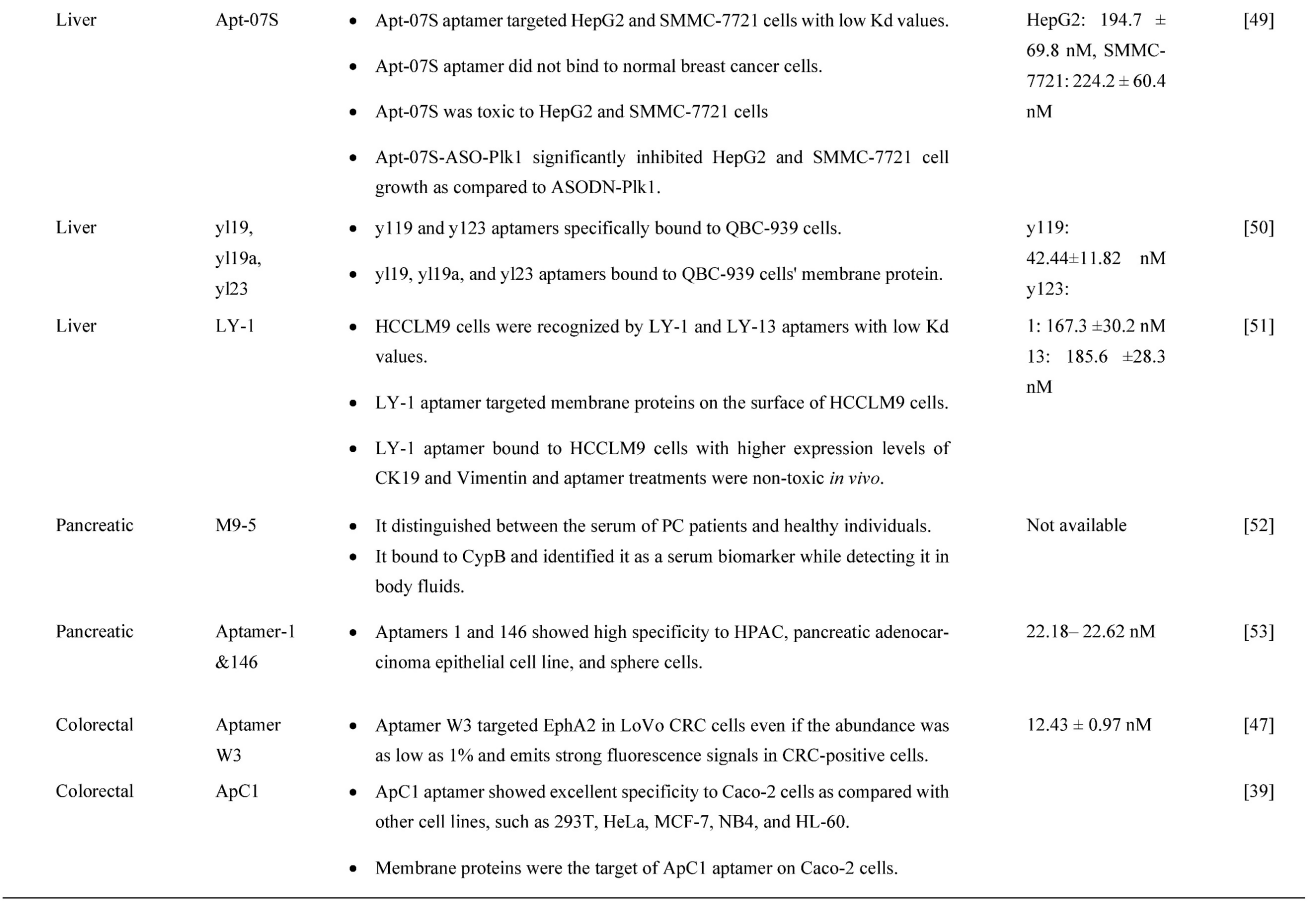

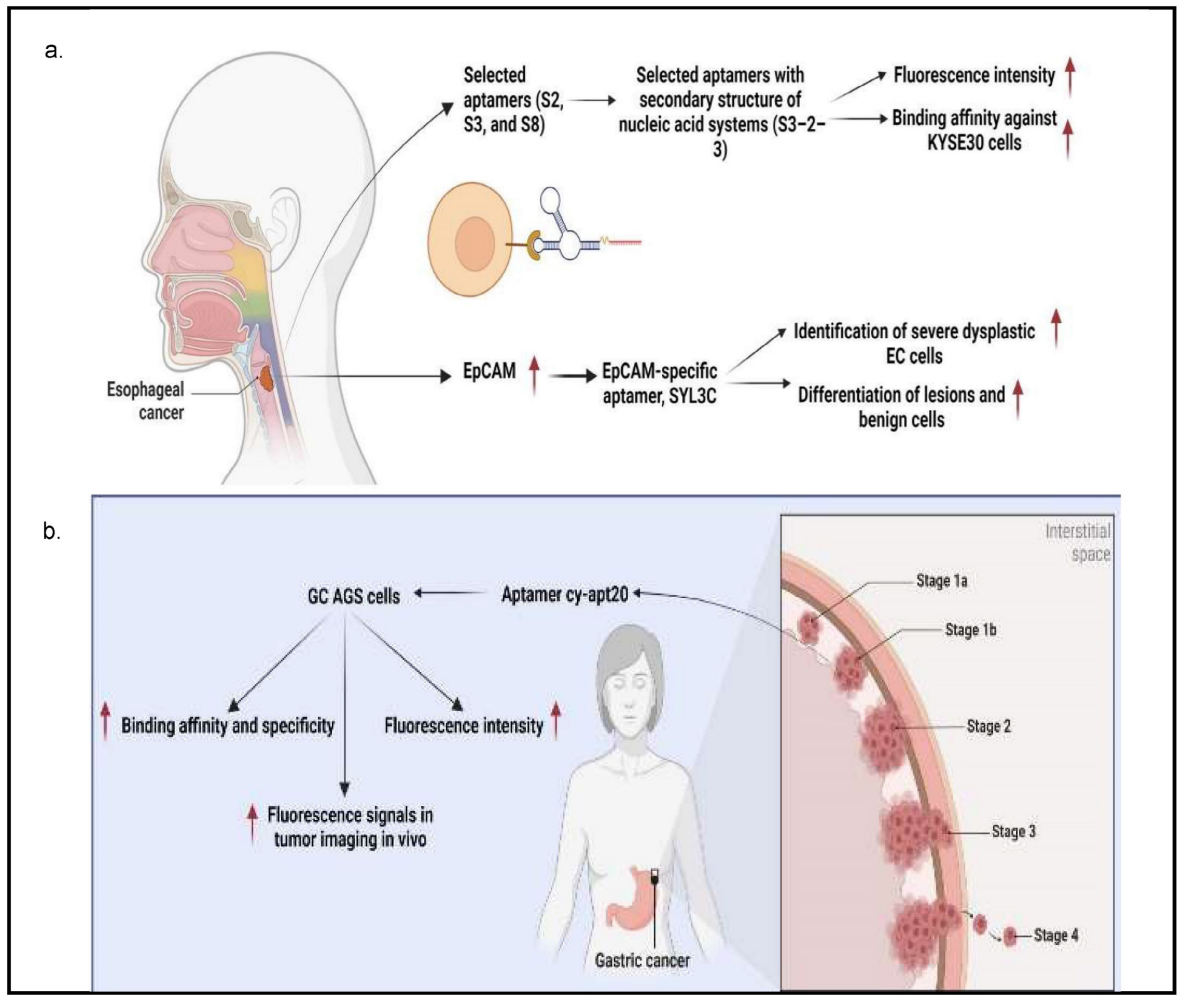

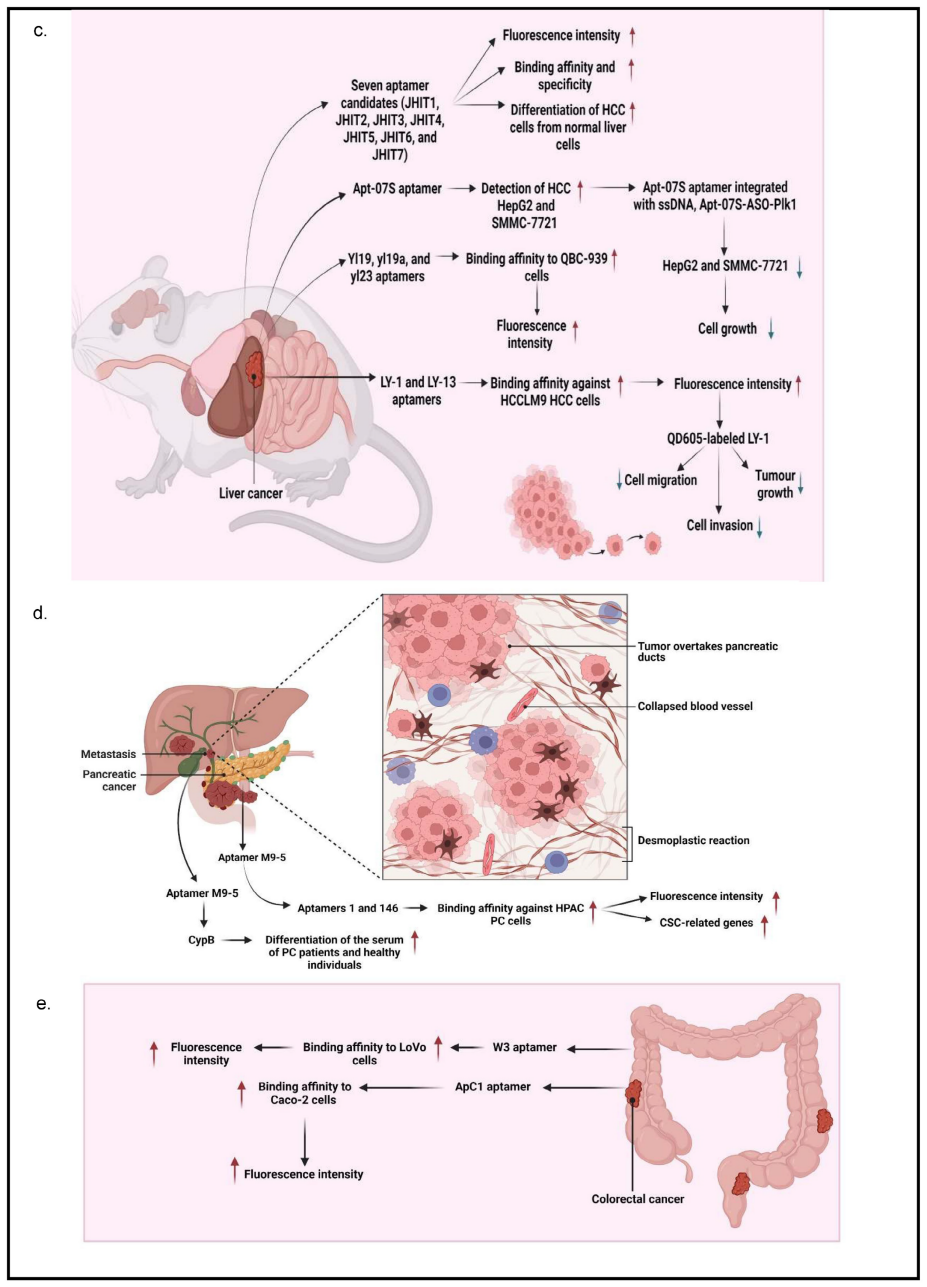
Aptamer-based specific detection of GI cancers. a. Specific detection in esophageal cancer; b. specific detection in gastric cancer; c. specific detection in liver cancer; d. specific detection in pancreatic cancer; and e. specific detection in colorectal cancer. This figure is created with biorender.com.

In a study by Li et al. 47, they studied a CRC-specific aptamer that specifically targeted ephrin type-A receptor 2 (EphA2), a protein involved in cell migration and invasion, in EphA2-expressing CRC cell line, LoVo cells. The binding affinity was reported to be 12.43 ± 0.97 nM kD proving a corresponding strong interaction between W3 and EphA2. This aptamer W3 successfully detected EphA2-expressing cells even though the presence of the target cells was as low as 1% and also emits strong fluorescence signals in CRC-positive cells when compared with adjacent CRC-negative cells. Using UNAfold software, Maimaitiyiming Y et al. 39 predicted that both forward and reverse primer binding sites participated in the important formation of hairpins for efficient binding of ApC1 aptamer to Caco-2 cells by truncation experiment. This finding also suggests that aptamers' fixed primer binding areas are critical for efficient target binding and that truncation is irrelevant to all aptamers. Both confocal microscopy and flow cytometry demonstrated that ApC1 aptamer only adhered to the Caco-2 cell membrane. Furthermore, aptamer ApC1 could rapidly and specifically internalize into Caco-2 cells because a strong fluorescence signal measured by flow cytometer was detected in the cytoplasm of Caco-2 cells as compared to other cancer cell lines, such as 293T leukemia, HeLa, MCF-7, NB4, and HL-60 cells. Lastly, pretreatment of aptamer ApC1 with trypsin indicated its target could be a Caco2 cell membrane protein. The above-mentioned studies have clearly indicated that aptamer-based detections and applications have exponentially increased over the years and has shown distinguished potential in GI cancers. Other than aptamers that contribute to diagnosis and prognosis, aptamers also have the potential to act as a drug delivery agent assisting in the process of chemotherapeutic drugs transport for cancer treatments. The summarization of aptamers as diagnostic or prognostic tools in GI cancers is mentioned in Table 2 and is summarized in Figure 3.

### 3.2. Aptamers as an Effective Drug Delivery System for Chemotherapy

Chemotherapy is one of the traditional clinical treatment methods for GI cancers, and it has many concerning side effects. Therefore, there is a need for an alternative method to reduce the side effects and improve the effectiveness of chemotherapy. Aptamers can be a useful tool to act as a drug delivery agent in transporting chemotherapeutic drugs to the target sites for higher treatment efficacy. For instance, a study by Ramezanpour M et al. 54 on chimera consisted of nucleolin-specific aptamer (aptNCL) and microRNA (miRNA) let-7d demonstrated potential therapeutic effects on GC cells using either covalent conjugation with SM (PEG)2 as a bifunctional crosslinker (product known as conjugate-1) or non-covalent conjugation with 19bp complementary sticky end sequences (product known as conjugate-2). Nucleolin is a protein that acts as a regulator of ribosomal DNA (rDNA) transcription and as a binding site for TNF-α inducing protein (Tipα), a recently discovered carcinogenic factor transcribed from Helicobacter pylori (H. pylori) strain from mouse GC cell line. It found that synergistic effects of the chimera on Januse kinase-2 (JAK-2) expression and activity, as measured by real-time PCR and enzyme-linked immunosorbent assay (ELISA) methods, could have a therapeutic effect on GC cells. Specifically, the chimera (i.e., conjugate-1, conjugate-2, aptNCL mixture of aptNCL and let-7d, or transfected let-7d) caused a substantial decrease in JAK-2 expression inMKN-45 GC cells after 24 hours treatment as compared to aptamer alone or untreated control condition (*p*=0.0001). Both conjugate-1 and transfected miRNA let-7d induced a considerable reduction in JAK-2 expression but not by conjugate-2 after 24 hours (p<0.01 and p=0.0001, respectively) and 48 hours (*p*=0.0001 and *p*<0.01, respectively), most likely due to enhanced stability. JAK-2 expression was unaffected by the aptNCL as compared to aptamer alone or untreated control condition. In contrast, under each of the chimera, the JAK-2 expression level of nucleolin-negative human derma fibroblast (HDF) cells (negative control) remained unchanged as compared to the control condition. Surprisingly, the transfected miRNA let-7d resulted in a significant reduction in JAK-2 expression in HDF cells (*p*=0.0001), demonstrating the non-specific effect of let-7d (lipofectamine) on both cancer and normal cells.

By means of internalization assay, Dua et al. 48 showed that Tetramethylrhodamine (TAMRA)-labeled SQ2 aptamer internalized into alkaline phosphatase placental-like 2 (ALPPL-2) expressing PC cells (i.e., Capan-1 and PANC-1 positive cells) within 30 minutes of incubation. This assay was carried out with RiboShredder, a harsh cocktail of ribonucleases, RNases, (to remove surface-bound aptamers) and 0.1% sodium azide (to inhibit internalization of any free TAMRA fluorochrome generated upon degradation of the RNA oligonucleotide). Intriguingly, treatment with endosomal inhibitors (MβCD) resulted in a 40% reduction in SQ2 binding to PANC-1 positive cells and nearly full loss of aptamer internalization, demonstrating that ALPPL-2-mediated cellular uptake of SQ2 is an energy-dependent mechanism. ALPPL-2 was mostly found in the cholesterol-rich fraction, and when cholesterol was removed from the plasma membrane, a portion of this glycosylphosphatidylinositol-anchored protein (GPI-AP) shed and the rest became incapable of caveolae-mediated endocytosis 48 suggesting that caveolae-mediated endocytosis is the most common mechanism for ALPPL-2 internalization in PANC-1 positive cells. Besides, the result further showed that SQ2 aptamer bound to ALPPL-2 with a Kd of 20 nM, which is low enough for targeted drug delivery to ALPPL-2 positive cells. The removal of the surface-bound aptamers revealed a significant amount of internalized SQ2 in both PANC-1 positive cells (26%) and Capan-1 positive cells (20%). Furthermore, the confocal microscopy observation identified the presence of streptavidin magnetic beads (2.8 mm in diameter) inside PANC-1 positive cells, indicating that SQ2 aptamer might effectively act as a cargo carrying NPs and other large molecules. The study also found that SQ2 carrying a 5-fluoro-2'-deoxyuridine (5FdU) pentamer (SQ2-5FdU) could inhibit Capan-1 cell growth by 40% and 52% at concentrations of 5 mM and 10 mM, respectively. Thus, SQ2 aptamer could successfully deliver the anticancer nucleoside drug, 5FdU to ALPPL-2-expressing PC cells without imposing toxicity on normal cells.

Gemcitabine, the gold standard drug for PC, has shown unsatisfactory therapeutic effects due to chemoresistance and low targeting of cancer cells. Ray P et al. 49 established an epidermal growth factor receptor-conjugated aptamer to deliver gemcitabine (E07-Gem polymer). It was purposely designed for binding to PC MiaPaCa-2 cells expressing EGFR. The results demonstrated that E07-GEM aptamer specifically recognized and bound to EGFR protein expressed on the cell surface of MiaPaCa-2 cells, indicating that this might be a viable approach to delivering gemcitabine to EGFR-expressing cells. Based on the internalization assay (Riboshredder assay), E07-Gem polymer was successfully internalized into MiaPaCa-2 cells. The process of endocytosis performed better at the physiological temperature of 37°C and was inhibited at 4^o^C. The percentage of E07-GEM internalized was higher at 100 nM (17%) versus 400 nM (6%). Upon treatment with E07-Gem polymer, MiaPaCa-2 cell viability was greatly inhibited as compared to mE07-Gem polymer or with the unescorted Gem polymer. Doxorubicin (Dox) is one of the most commonly used chemotherapy drugs for many cancers. A study by Wu et al. 40 determined that aptamer XQ-2d binds specifically to CD71, an overexpressed protein in PC. An XQ-2d-based complex for loading Dox was used to develop an XQ-2d-mediated targeted treatment for pancreatic cancer. A chimera was generated with this aptamer, XQ-2d-sd3-DOX, to investigate the inhibitory effect of this chimera sequence in PC. The chimera did exhibit significant suppression of proliferation in PL45 cells, pancreatic adenocarcinoma epithelial cell line, showing the potentiality of molecular targeted therapy.

According to a study by Yao F, et al. 50, fluorescent-labeled Apt-HJ aptamer exhibited a high affinity toward colon cancer cells (nucleolin-positive CT26 cells), as evidenced by considerably higher fluorescence signals in flow cytometry detection. This study evaluated the drug loading capacity of Apt-HJ after mixing with Dox at increasing molar ratios due to Dox intercalated into double-stranded DNA (dsDNA) structures to form a Dox-DNA complex while free Dox emitted a red fluorescence that will be quenched when Dox intercalated into dsDNA. The findings show that the fluorescence was almost quenched when the molar ratio of Apt-HJ to Dox increased to 1:17, indicating that most Dox molecules intercalated into the DNA structure of Apt-HJ. After treating with Apt-HJ-Dox, red fluorescence was observed in CT26 cells but slightly in Chinese hamster ovary (CHO) cells as observed under confocal microscopy, directly indicating that Apt-HJ-Dox was delivered into CT26 cells but not CHO control cells. Interestingly, the result also showed that Apt-HJ-Dox killed CT26 cancer cells (55.14% ± 4.34%) efficiently while also reducing damage to normal CHO control cells (80.72% ± 5.71%,) as compared to free Dox. Apt-HJ-Dox exhibited a substantially larger half maximal inhibitory concentration, IC_50_, for CHO cells than free Dox (8.481 M vs. 2.579 M), demonstrating that Apt-HJ-Dox induced preferential cytotoxicity against CT26 cells while reducing toxicity toward control cells. Furthermore, a similar result was also observed in the *in vivo* study in which Apt-HJ-Dox demonstrated pronounced antitumor activity in mice carrying CT26 tumors as compared to free Dox and Apt-HJ. The average tumor volume of mice treated with Apt-HJ-Dox was five times smaller than that of the control group, with no additional weight loss observed in the Apt-HJ-Dox group.

Apart from this, Nejabat M et al. 51 showed that covalent conjugation of DOX-encapsulated hollow mesoporous silica NPs (HMSNs) coated with acetylated carboxymethyl cellulose NPs to AS1411 aptamer (Apt-NP-DOX) led to a specific delivery of Dox to nucleolin-overexpressed MCF-7 cells. Based on flow cytometry analysis, the cellular uptake efficiency of Apt-NP-DOX was higher in nucleolin-overexpressed MCF-7 cells than conventional Dox, HMNS/AcCMC-DOX (NP-DOX). Additionally, no significant difference was observed between the cellular uptake of targeted and non-targeted formulations in nucleolin-negative CHO cells. However, nucleolin positive cell lines (i.e., MCF-7 cells and C26 colon cancer cells) demonstrated superior toxicity when treated with Apt-NP-DOX than NP-DOX. However, there no significant difference in cytotoxicity was observed between Apt-NP-DOX and NP-DOX in CHO cells. Conventional DOX showed higher cytotoxicity in all cell lines tested, as it has a small size and can freely enter cells via simple diffusion. Similarly, a single dosage of Apt-NP-DOX dramatically reduced tumor growth in BALB/C mice transplanted with C26 cells as compared to free Dox- and NP-Dox-treated groups. Interestingly, mice administered with Apt-NP-DOX did not cause body weight loss and death when compared to mice administered with NP-DOX and free Dox. The types of chemotherapy treatments and their drug delivery methods for GC, PC and CRCs are summarized in Table 3.

## 4. Aptamers Bind to Different Molecular Targets as a Potential Cancer Treatment

Aptamers specifically bind to molecular targets in a cell, and they vary between surface proteins, peptides, toxins, and small molecules. They are selectively identified to distinguish cancer-related proteins and cells involved in tumorigenesis 52. Identifying cancer biomarkers or molecular targets is crucial for improving cancer treatments, such as chemotherapeutic drug delivery, molecular imaging, and targeted molecular therapy. Many studies have identified various types of molecular targets for different types of GI cancer to be targeted by aptamers as potential cancer therapy. In this section, the different types of molecular targets targeted by aptamers as potential cancer treatments are listed for several types of GI cancers, including esophageal, gastric, liver, pancreatic, and colorectal.

### 4.1. Esophageal Cancer

EC is listed as one of the most common types of GI cancer, and it is ranked the ninth most prevalent cancer worldwide. It consists of two subtypes, namely esophageal adenocarcinoma (EAC) and ESCC 32. Although ESCC contributes to about 90% of total EC cases, EAC affiliates with higher mortality when compared to ESCC 53. The lack of efficient treatments due to poor diagnosis and prognosis in EC causes this dismaying outcome on a global scale 55. EC often pervades and metastasizes into the adjacent organs prior to early detection, and this causes the existing cancer treatments to be less responsive to cancer cells 44. Currently, the most common conventional treatments for EC are chemotherapy and curative surgery 32. Recently, targeted therapy has been reflecting tremendous progress in improving the diagnosis and prognosis of EC by applying aptamers targeting molecular targets 44.

Chen et al. 32 established three aptamers (i.e., S2, S3, and S8) and an ultrashort aptamer S3-2-3 using the cell-SELEX technology. These aptamers successfully bound to membrane proteins in ESCC tissues. The same study confirmed that the aptamers bound to membrane proteins in their selected KYSE150 cells but did not mention any specific membrane proteins. Because the target of the aptamers were membrane proteins, thus a decline in fluorescence signals of the aptamers was observed after the proteins were degraded with trypsin and proteinase K as compared to the untreated control. Apart from these, SOX2 protein, also known as sex-determining region Y)-box 2 (SRY), is also a molecular target of ESCC cells, which has been shown to regulate the growth and maintenance of esophagus stem cells and overexpressed in different types of cancer malignancies contributing to the initiation, invasion, and migration. Liu et al. generated a peptide aptamer, P42, and a synthetic peptide aptamer, sP42, to specifically target SOX2 protein and suppress the malignant tendencies by inhibiting proliferation, migration, and invasion in ESCC cells. Treatment of both P42 and sP42 aptamers successfully repressed tumor growth and proliferation, as shown in xenograft mouse and zebrafish models. The ectopic expression of P42 aptamers significantly reduced about 86% of the tumor mass weight and inhibited the metastasis of ESCC cells in zebrafishes 55. A more recent study by Zhang et al. 56 developed a few aptamers to detect ESCC-positive cells with cell-SELEX technology. The most successful aptamer to detect the ESCC-positive cells was an aptamer labelled A2. This study reports that the molecular target for aptamer A2 is a cell surface receptor protein, integrin beta-1 (integrin β1), also commonly known as the CD29 protein. This aptamer contributed to the clinical imaging by emitting strong fluorescence signals when detecting ESCC-positive cells with adjacent cells. The aptamer A2 also showed potential to be a great drug delivery agent, it suppressed tumor growth in xenograft mouse models.

### 4.2. Gastric Cancer

GC was ranked the third most common by World Health Organization in 2015 19. It is known as one of the aggressive malignancies because of its 5-year survival rate of less than 25% and is only identified in patients once it has developed to an advanced stage 33. The most prevalent treatments for GC are curative and adjuvant treatments. Although many improvements have been made in these treatments, the lack of early detection and vigorous advancement of tumors hinder their significant development 19, 33. Therefore, much research has been done to identify molecular targets in targeted therapy and determine the best aptamer as a detection tool using *in vitro* screening 19. Watanabe et al. 57 and Daei et al. 58 discovered that nucleolin could be a molecular target for GC-positive cells. They found that nucleolin was overexpressed in GC cells; thus, they generated aptamers specifically bound to nucleolin-positive cells instead of nucleolin-negative cells 57, 58. Watanabe et al. used a well-known anticancer aptamer, AS1411, which surpassed the Phase I clinical trial to treat acute myeloid leukemia and kidney renal cell carcinoma. AS1411 aptamer specifically bound to nucleolin and triggered inhibition of GC cell growth and induced apoptosis. GC cell growth inhibition was determined with a dose-dependent addition of aptamers. MKN-1, a GC cell line with a low presence of nucleolin, showed a similar rate of cell growth inhibition as compared to MKN-74, a GC cell line with a high presence of nucleolin 57. On the other hand, Daei et al. generated a conjugate of the AS1411 aptamer, known as an aptNCL-let-7d conjugate, bound to nucleolin to release let-7d, a tumor suppressor, into GC cells. Both aptNCL and aptNCL-let-7d conjugate significantly inhibited GC cell proliferation as compared to their counterparts for both 24 hours and 48 hours of treatment. In addition, aptNCL-let-7d triggered high production of let-7d in GC cells and significantly reduced tumorigenesis. It has been proved that let-7d has a role in anticancer activity through targeting oncogenes, such as JAK2/STAT3, RAS, high mobility group A2 (HMGA2), and cellular Myc (c-MYC), which are crucial for cancer progression 58.

JAK-2 protein plays a prominent role in regulating growth, invasion, and apoptosis in GC cells. It is also associated with the signal transducer and activator of the transcription (STAT) pathway contributing to cancer cell growth and proliferation. Ramezanpour et al. 54 attempted to use a nucleolin-specific aptamer with miRNA let-7d chimera to evaluate the effect on the expression of JAK-2. After treating JAK-2-positive GC cells with NCL-Apt-miRNA let-7d chimera, it was found that JAK-2 expression levels were significantly downregulated. This finding indicates that JAK-2 can be used as a molecular target in GC cells, and this specific chimera has the potential as a targeted therapy for GC. Furthermore, both control counterparts, NCL-Apt and miRNA let-7d aptamer, did not show a promising effect on JAK-2 expression in GC cells, implying that the combination of NCL-Apt and miRNA let-7d showed a great response in inhibiting JAK-2 activity 54.

Besides, several RNA aptamers have been designed to specifically bind to other identified molecular targets in GC, including CEA A01 and A02; cancer antigen 50 (CA50), A01, and A02; and CA72 A01 and A02. CEA is a surface protein that contributes to cell adhesion and intracellular signaling, while CA50 is a monoclonal antibody that has a prominent presence in GI cancers. CA72-4 is a tumor-associated antigen that is more sensitive than CEA. The combination of aptamers binding to their respective type of six antigens reduced GC cell viability and proliferation 19. Lastly, aptamers have targeted another molecular target: ERBB-2/HER2. ERBB-2/HER2 is a receptor protein that has a crucial role in epitheliogenesis and has been shown to be overexpressed in GC cells. Mahlknecht et al. selected a trimeric aptamer, an aptamer with the trimeric version of the identical 14 selected nucleotides, to treat ERBB-2 positive GC cells. The treatment of trimeric aptamer targeting ERBB-2 significantly reduced cell growth and proliferation *in vitro* and *in vivo*. The study further showed that when the trimeric aptamer was introduced to ERBB-2-positive cells, the cell viability was suppressed successfully. As for the tumor growth in mice, the aptamer inhibited the growth of tumor mass as compared to the antibody treatment 59.

### 4.3. Liver cancer

LC is the fifth most common cancer and does have one of the highest mortality rates 37. HCC is the most common type of LC; it has invasive and metastatic properties that fail many common treatment options 36. Due to these properties, HCC is often unable to be diagnosed early in patients and is most likely to reoccur quickly. Despite having a low survival rate and high metastasis rate, the common treatments used for LC were curative surgery, chemotherapy, or locoregional therapies 37. Therefore, there is an urgent need to search for a treatment that does not cause high toxicity and specifically targets LC cells 60.

AFP is the first tumor marker identified in LC and possesses an important role in diagnosing and treating LC. It is a major serum glycoprotein and is highly expressed in HCC cells. In a study by Lee and Lee 61, a specific aptamer was created to target AFP in HCC cells to regress the AFP overexpression and to activate cell growth inhibition to prevent HCC proliferation. Thus, they developed group I RNA aptamer, and it specifically bound to AFP and successfully inhibited AFP-mediated cell proliferation and overexpression of oncogene mRNA 61. Similarly, Dong et al. also developed AP273 aptamer binding to AFP in AFP-expressing HCC cells. It was shown that AP273 aptamer could inhibit HCC cell migration and invasion after the cells were transfected with AP273 62. Both studies have proved that AFP is an ideal molecular target for LC. It has been reported that only 30-40% of LC are AFP-negative; thus, a different molecular target is required for HCC. Heterogeneous nuclear ribonucleoprotein A1 (hnRNP A1) has been reported to contribute to mutant p53 expression that influences cell proliferation and oncogenic properties. Cell proliferation and migration regressed when hnRNP A1 are downregulated with siRNA-specific for hnRNP A1, siRNPA1, in HCC cells. Besides, a new aptamer targeting hnRNP A1 was also developed by Li et al. 63. In this study, high levels of hnRNP A1 expression were detected in HCC cells using hnRNP A1-specific ssDNA aptamer, BC15, because it was proven to target hnRNP A1 specifically. This aptamer also inhibited tumor growth in a xenograft model transplanted with HepG2 cells stably expressing an enhanced green fluorescent protein, HepG2-EGFP 63.

Anticancer drugs are often used in treating LC, particularly Dox. Due to the fact that Dox exerts significant side effects on the heart and liver, thus a more efficient anticancer drug delivery method is required to reduce toxicity and achieve active targeting in the tumor simultaneously. Zhou et al. discovered an aptamer-based drug delivery agent called CD133-apt-Dox. This aptamer was designed to target CD133. CD133 is highly expressed in LC, and it showed that CD133-apt is ideally bound to CD133-positive cells. The cellular uptake of CD133-apt-Dox was higher and more efficient than free Dox. Furthermore, CD133-apt-Dox also demonstrated stronger cytotoxicity against HCC cells and organoids, affected and impaired stem-likeness causing drug resistance in mice, and blocked CD133-mediated oncogenic pathway by reducing tumor growth and proliferation. These findings indicate that CD133 can be used as a molecular target for LC, and CD133-apt can be used as a drug delivery agent for Dox 64. Similarly, in the most recent study by Yin et al. 65 it was reported that CD133 was the molecular target for CD133 aptamer-DOX conjugate as a drug delivery agent for a HCC cell line. This aptamer specifically bound to CD133-positive expressing cell line when compared to a CD133-negative expressing cell line and increased the uptake and retention of Dox in the HCC cell line. CD133 aptamer-DOX conjugate was proven to promote apoptosis and suppressed autophagy to enhance the efficiency of Dox treatment. A study done by Liu et al. 60 successfully designed an aptamer-mediated gene delivery to LC cells by using highly expressed EpCAM as the LC cell target. Ad5-PTEN is a recombinant adenovirus expressing the tumor-suppressor gene of PTEN that has anticancer properties in reducing tumor proliferation, invasion, and migration. This study evaluated the delivery efficiency of the Ad5-PTEN gene to EpCAM-conjugated aptamers, EpDT3-mediated Ad5-PTEN (EpDT3-PEG-Ad5-PTEN, EPAP). EPAP treatment inhibited the migration capacity of EpCAM-positive LC cells and repressed their metastasis. Therefore, EPAP can be a good alternative cancer therapy for LC 60. Dong et al. identified that GPC3, a cellular membrane proteoglycan that has been shown to overexpress in HCC, is another molecular target that has been conjugated with an aptamer for treating HCC. In fact, GPC3 is highly expressed in many HCC cells via antibody-based histopathological systems and has been identified as a tumor biomarker 38. Another study on HCC evaluated the potential of an aptamer in targeting HCC cells and being a drug delivery agent. The result showed that the aptamer with an integrated ssDNA (Apt-07S-ASO-Plk1) bound to a *Plk1*. *Plk1* is a gene that is involved in cell proliferation and is highly expressed in HCC cells. *Plk1* gene silencing using ASODN in this selected aptamer could enhance HCC cell growth inhibition and decrease *Plk1* expression 37.

### 4.4. Pancreatic cancer

Many researchers have utilized different targets of aptamers to develop agents that can be used for the targeted treatment in PC. A secretory protein termed pancreatic adenocarcinoma up-regulated factor (PAUF) is widely produced in PC, which has been implicated in PC cell proliferation and metastasis. In view of this, Kim et al. developed and tested a 2'-fluoropyrimidine modified RNA aptamer (P12FR2) to target PAUF. P12FR2 bound to PAUF with an apparent Kd of 77 nM. In a wound-healing experiment, P12FR2 aptamer suppressed PAUF-induced PANC-1 cell migration. Furthermore, in an *in vivo* xenograft model transplanted with CFPAC-1 PC cells, intraperitoneal injection of P12FR2 significantly reduced tumor growth by up to 60% without inducing weight loss in the treated mice. They found that the aptamer P12FR2 had the potential to be useful in the treatment of PC 66. Despite current chemotherapeutic agents and molecular target inhibitors, the 5-year survival rate of PDAC is still depressing. More appropriate multi-drug approaches are thus mandatory to flatten the rising prevalence rate of PDAC. Yoon et al. 41 proposed a novel aptamer-based strategy for delivering targeted molecular therapy in advanced PDAC patients who failed to respond to other treatments. The upregulation of the transcriptional factor CCAAT/enhancer-binding protein (C/EBP), known for its anti-proliferative properties, was reported as a novel means of providing anti-tumorigenic therapy in PDAC. For the creation of a cell type-specific delivery vehicle, small activating RNA (saRNA) duplexes were designed to promote C/EBP expression, followed by attaching to PDAC specific 2′-Fluropyrimidine RNA aptamers (2′F-RNA) - P19 and P1. Intriguingly, P19- and P1-C/EBP-saRNA conjugates drastically reduced PDAC cell proliferation while increasing C/EBP expression. Similarly, it was found that injections of saRNA/aptamer conjugates into the tail veins of xenograft mice, either transplanted with PANC-1 or gemcitabine-resistant AsPC-1 cells, reduced tumor size with no significant side effects 41.

Dua et al. 67 identified an aptamer SQ-2 that could recognize PC cells with a high specificity using a selection strategy that could generate aptamers for two PC cell lines in one selection scheme. ALPPL-2, an oncofetal protein, was identified as the target of SQ-2. *ALPPL-2* gene silencing via RNA interference revealed new tumor-associated roles of this protein in PC cell proliferation and invasion. Furthermore, in a different study, the target of aptamer P15 was identified as the intermediate filament vimentin, a biomarker of epithelial-mesenchymal transition (EMT), which is an intracellular protein but is specifically expressed on the plasma membrane of cancer cells, according to the results of an unbiased proteomic mass spectrometry approach. Tumor cell metastasis assays were performed *in vitro* because EMT plays a critical role in the transition of cancer cells to invasive cells. The tumor metastasis of P15-treated PC cells was significantly reduced. EMT-related gene expression analysis was used to identify differentially expressed genes to investigate the downstream effects of P15 (DEG). The expression of matrix metallopeptidase 3 (MMP3), which is involved in cancer invasion, was downregulated in P15-treated cells among five DEGs. P15 binding to cell surface vimentin inhibited tumor cell invasion and was associated with lower MMP3 expression, implying that P15 could be used as an anti-metastatic therapy in PC 68.

G-protein-coupled cholecystokinin B receptor (CCKBR) is expressed constitutively in PDAC, which plays a crucial role in cell proliferation and apoptosis. It has been proven that when CCKBR is downregulated, cell proliferation decreases, and cell apoptosis increases 69. Clawson et al. 70 demonstrated DNA aptamers' characterization and targeting efficiency binding to CCKBR. A pool of DNA aptamers was discovered using dual SELEX selection against ''exposed" CCKBR peptides and CCKBR-expressing PDAC cells. Eight DNA aptamers were chosen for initial characterization after selection based on projected structures and features. The DNA aptamers bound exclusively to CCKBR and found that they suppressed cancer cell proliferation and reduced PDAC cell growth. Only one aptamer, dubbed AP1153, was chosen for further binding and localization experiments because it possessed the most reliable predicted secondary structure. Clawson et al. discovered that AP1153 aptamer did not activate CCKBR signaling pathways, and AP1153 aptamer was internalized by PDAC cells in a receptor-mediated manner, as visualized using three-dimensional confocal microscopy. The binding affinity of AP1153 aptamer was found to be 15 pM. The bioconjugation of AP1153 aptamer to the surface of fluorescent NPs made NP delivery to PDAC tumors much easier. This AP1153-targeted NP delivery system's selectivity improved the early detection of PDAC lesions and enhanced chemotherapeutic treatments for PDAC patients 70. In PC cells, increased expression of high mobility group A (HMGA) protein is linked to resistance to gemcitabine 71. The use of HMGA-targeted AT-rich phosphorothioate DNA (AT-sDNA) aptamers to reduce HMGA carcinogenic activity was established by Watanabe et al. 71. After treatment with gemcitabine, AsPC-1 and MiaPaca-2 cell transfected with AT-sDNA was evaluated. Both cells showed a significant increase in cell death when AT-sDNA-transfected cells were compared to non-AT-rich sDNA-treated cells. The findings suggested that HMGA targeted DNA aptamers could be used to improve chemotherapy efficacy in PC treatment 71. Lastly, a different study showed that PC cells also highly expresses CD71 providing potential as a molecular target for combating PC. In this study, they generate an aptamer that is specific to CD71, paclitaxel (PTX)-modified polydopamine (PDA) nanospheres with the conjugation of peptidomimetic AV3 called Apt-PDA@PTX/AV3 bioconjugates. *In vitro*, this aptamer specifically inhibited cell survival and promoted cell apoptosis within PC-expressing cells. In vivo, this aptamer successfully showed results in inhibiting desmoplastic stroma, a result that corresponds with a poor prognosis, showing great potential in efficiently suppressing PC cells 72.

### 4.5. Colorectal Cancer

CRC is one of the most frequent cancers, with rates of diagnosis and incidence steadily rising 73. CEA is overexpressed in CRC patients and is linked to cell adhesion, anoikic resistance, and metastasis to the liver 74. 5-fluorouracil, 5-FU, is one of the most frequently used chemotherapeutic agents to treat CRC 75. However, chemoresistance is a severe issue with 5-FU therapy because it causes CEA overexpression, subsequently causing 5-FU resistance. In a mouse model, a CEA-specific RNA aptamer was found to suppress CRC cells' hepatic metastasis. In chemoresistant LS174T CRC cells, Lee and Lee 76 investigated if shielding CEA with CEA aptamer could improve 5-FU sensitivity. When compared to cells treated with 5-FU alone (IC50 31.46 M), CEA aptamer sensitized 5-FU-resistant LS174T cells to 5-FU by more than 5-fold (IC_50_ of 5.995 M). Furthermore, in a xenograft mouse model, CEA aptamer coupled with 5-FU synergistically reduced the growth of chemoresistant tumors. In the 5-FU-resistant LS174T and xenograft tumors, a combination of 5-FU and CEA aptamer increased caspase-8 activity via aptamer-mediated disruption of CEA interaction with death receptor 5. CEA-specific aptamer increased 5-FU sensitivity in both *in vitro* and *in vivo* studies, thus constituting a novel 5-FU adjuvant to overcome chemoresistance in CRC patients 76.

One of the most significant drawbacks in cancer treatment is the development of chemoresistance and the failure to eliminate CSCs 77. For future effective management of cancer, novel molecular therapeutic methods to overcome these constraints are urgently needed. AlShamaileh et al. 78 demonstrated that EpCAM-aptamer-guided survivin RNAi successfully downregulated survivin in CRC cells and a xenograft mouse model. Besides, the aptamer-guided survivin RNAi improved the sensitivity to 5-FU or oxaliplatin in CRC stem cells, increased apoptosis, inhibited tumor growth, and improved the overall survival of mice bearing xenograft CRC when used in combination with conventional chemotherapeutic agents. The findings suggest that survivin is one of the essential factors in CRC stem cells' innate chemoresistance. As a result, aptamer-mediated survivin targeting in CSCs combined with chemotherapeutic agents could offer a novel way to improve treatment outcomes in oncology clinics 78. MiRNAs, a tumor suppressor gene, are overexpressed in CRC and have been reported to prevent or delay cancer progression 79. However, using miRNAs as therapeutic agents needs to resolve several challenges, including a rapid breakdown in plasma, low absorption, and off-target effects 80. For the targeted therapy of CRC, Zhao et al. 80 established a cationic liposome-based nanoparticle loaded with miR-139-5p (miR-139-5p-HSPC/DOTAP/Chol/DSPE-PEG2000-COOH NPs) known as MNPs and miR-19-5p surface-decorated with EpCAM aptamer (miR-139-5p-EpCAM Apt-HSPC/DOTAP/Chol/DSPE-PEG2000-COOH) known as MANPs, which were 136.1 ± 4.8 nm and 150.3 ± 8.8 nm in size, with a round form and functional dispersion capabilities. MANPs significantly reduced CRC cell proliferation, migration, and invasion. The study also showed that MANPs could be taken up and targeted in *vitro* and *in vivo*. It also found that MANPs reduced the development of HCT8 cells and displayed a strong tumor-suppressive effect in subcutaneous HCT8 colorectal carcinoma mice 80. The findings show that MANPs is a good carrier for delivering therapeutic miRNAs to CRC patients. MANPs prove to be an efficient targeted therapy by inducing anticancer activities. Another recent study 81 reported an aptamer with magnetic mesoporous silica nanoparticles with EpCAM showed efficient delivery of 5-FU to CRC cancer cells. This aptamer, Apt-PEG-Au-NPs@5-FU, targets EpCAM in EpCAM-expressing CRC cell line, induces cell apoptosis *in vitro* and inhibits tumor growth *in vivo*. Moreover, this aptamer increases uptake of the chemotherapeutic drug showing great potential is efficient drug delivery of 5-FU in CRC.

**Figure 4:**
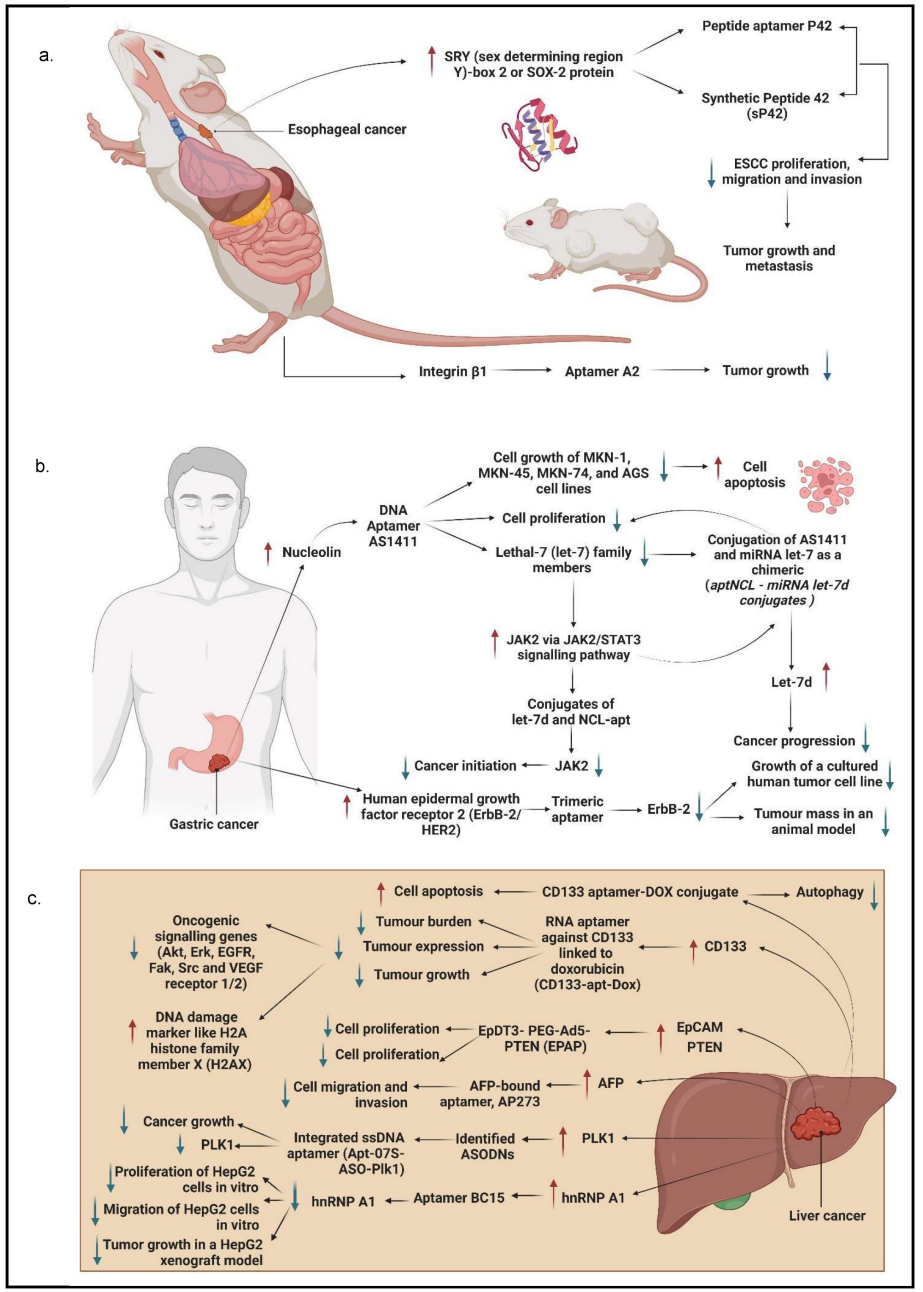

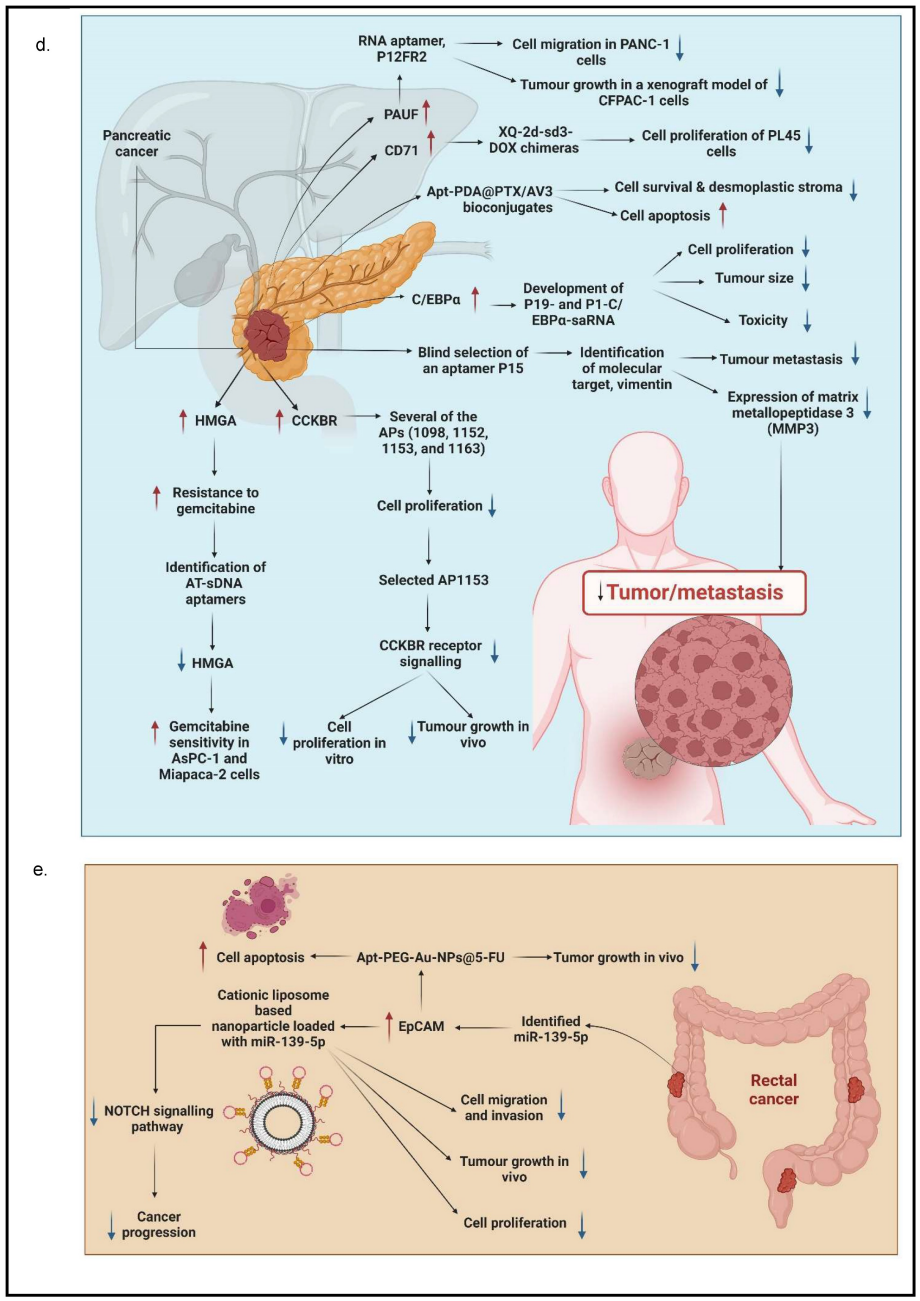
Different types of molecular targets targeted by aptamers as potential cancer treatments. a. Potential aptamer-based treatments in esophageal cancer; b. potential aptamer-based treatments in gastric cancer; c. potential aptamer-based treatments in liver cancer; d. potential aptamer-based treatments in pancreatic cancer; and the potential aptamer-based treatments in colorectal cancer. This figure is created with biorender.com.

Lastly, S100P functions as a Ca^2+^ binding protein in calcium-dependent signal transduction pathways involved in various biological activities. It has been shown to play a crucial role in promoting cell proliferation, invasion, and metastasis in CRC tissues and is overexpressed in CRC 82. As a result, S100P could be a promising molecular target for CRC treatment. For instance, Sun et al. 82 used the SELEX technique and high-throughput sequencing to create a new DNA aptamer targeting S100P (AptS100P-1). AptS100P-1 had a significant affinity for S100P protein, as evidenced in the binding experiment. AptS100P-1 was also relatively stable in a cell culture system and could be utilized to detect S100P via flow cytometry, dot blot assays, and fluorescence microscopy. Besides, it also attached and inhibited CRC cell growth both *in vitro* and *in vivo*. AptS100P-1 also prevented S100P-expressing CRC cells from migrating and transitioning from epithelial to mesenchymal cells by suppressing the expression of EMT protein and overturning the EMT process. The findings indicate that using AptS100P-1 to target S100P could be a viable treatment strategy for CRC 82. Figure 4 below mentions the different types of molecular targets targeted by aptamers as potential cancer treatments in GI cancers and Table 4 below mentions all the relevant functions and molecular mechanisms of aptamers associated with molecular targets in GI cancers.

## 5. Current Applications of Aptamers in Clinical Settings for GI Cancer Treatment

The number of clinical trials evaluating aptamer-based drugs is increasing due to their great potential as prognostic, diagnostic, and therapeutic tools. Therapeutic aptamers are promising agents for treating GI cancers in the clinical setting because they can efficiently differentiate between normal and tumor tissues, as well as between different tumor types and can exert better tumor-killing effects 83. In preclinical trials, few therapeutic aptamers with antagonistic functions have shown high affinity, specificity, and inhibitory effects against GI cancers. Additionally, preclinical studies also validate their efficacies and safety profiles 84. Up to now, mounting evidence has reported that two anticancer aptamers have been successfully tested in clinical trials, namely AS1411 and NOX-A12 aptamers. AS1411, a nucleolin-targeting DNA aptamer, is the first aptamer that has made a successful transition to clinical trials for cancer treatment. It shows an effective inhibition against nucleolin function in cancer cells and displays an immense anti-proliferative effect in solid tumors 85 and renal cell carcinoma 86, 87. While NOX-A12 is a second 45-nt L-ribose-based RNA therapeutic aptamer, which is an antagonist for chemokine CXCL12 (or SDF1). It is also known as a Spiegelmer because of its mirror-image oligonucleotides that have high resistance to nucleases. In preclinical trials, NOX-A12 effectively prevented chemotaxis of hematologic malignancies 88 and is currently in clinical trials testing on CRC or PC 89. On the other hand, a clinical trial study with 40 participants for PC has completed its trial for a cancer stem cell specific aptamer to study the prognostic role detecting the cancer stem cells but no results or articles has been published based on this study 90. To date, the literature shows that the research on RNA and DNA aptamers has expeditiously increased, but the development of clinically useful aptamer therapeutics is growing slowly. This section highlights the current advances and applications of RNA, DNA, and conjugation of aptamers in research and clinical trials.

### 5.1. RNA aptamers

RNA aptamers have unique advantages for therapeutic and diagnostic applications because of their high binding affinity and specificity for cancer treatment. To date, there are still few RNA aptamers under preclinical and clinical trials for cancer treatment. As mentioned above, NOX-A12 is a well-established RNA aptamer in clinical trials, and, currently, clinicians are testing its anti-proliferative effectiveness on CRC or PC 91. The above findings imply that research on RNA aptamers is still in its infancy, where it requires further understanding of nucleic acid chemistry target interaction, tissue distribution, and pharmacokinetics. Therefore, only a few RNA aptamers have reached the market after several challenges.

### 5.2. DNA aptamers

DNA aptamers are well explored as compared to RNA aptamers because of their stability which allows them to be used as a diagnostic tool. As discussed earlier, AS1411 is a well-known first nucleolin-targeting DNA aptamer and is used for cancer treatment in clinical trials. It shows promising anti-proliferative activity against different cancer types, such as colon, liver, lung, breast, and cervical 92. A clinical trial in its early phase is studying Sgc8, a DNA aptamer, that is being used to measure the radiation exposure via radiation therapy of 68Ga-Sgc8, a protein, in healthy participants and to study its diagnostic value in colorectal cancer patients but no results are available 93. These preclinical and clinical results suggest that DNA aptamers are highly in demand and have the potential to be used in GI cancer identification and treatment.

### 5.3. Conjugation of Aptamers

Aptamers are broadly used in cancer research due to their specificity, and flexibility, and can be structurally modified in order to conjugate with various molecules (e.g., fluorescent agents, NPs, quantum dots, etc.) to increase their targeting efficiency for drug delivery to cancerous tissues. Even though aptamer drug conjugates are well applied in cancer research, it has not progressed to clinical trials 94. Although there are several promising research investigating on potential conjugation of aptamers with various molecules, such as fluorescent agents, NPs, quantum dots, etc., for cancer therapy, much work needs to be done prior to translating into clinical practice, including reducing the toxicity of the conjugate and improving the binding specificity and sensitivity to target cells.

## 6. Conclusions and Future Perspectives

Aptamers are discovered via cell SELEX technology and are currently and widely used in cancer diagnosis, as a prognostic tool, and as potential biomarker discoveries. They are better alternatives to antibodies for their low toxicity, easy production, and no limitations against their targets' size. Cell SELEX technology is currently the most efficient procedure for selecting specific aptamers that bind to molecular targets or membrane proteins in GI cancers. However, GI cancers are more prevalent in Asia, primarily due to the lack of diagnostic and prognostic tools. Aptamers bind to cancer biomarkers, potentially improving the diagnosis, prognosis, and treatments for GI cancers. They are often known for their versatility because they can be easily modified specifically to their aimed targets. Besides, they can be generated specifically to target biomarkers in various secondary or tertiary structures. Their application can detect and differentiate cancer cells with specific affinity and act as a drug delivery system for chemotherapy treatments. There are a few types of aptamers, including RNA, DNA, and conjugate aptamers. DNA aptamers are widely studied for their stability in cancer treatments when compared to RNA aptamers, although RNA aptamers are discovered to have high specificity and binding affinity in cancer-related studies. On the other hand, conjugated aptamers are favored in chemotherapeutic treatments for their increased target efficiency as drug delivery agents. RNA aptamers are easily synthesized but have the possibility of breaking down easily in our system, and conjugated aptamers need more studies done to determine their toxicity. In this review, we have briefly discussed the generation of aptamers, the Cell SELEX process, types of aptamers, several different GI cancers associated molecular targets, the application of aptamers as a cancer biomarker, diagnostic or prognostic tool, and chemotherapeutic drug delivery agents. The GI cancers we reviewed were esophageal, gastric, liver, pancreatic and CRCs. In summary, the identified molecular targets for EC it was SOX-2 protein, for GC they were nucleolin, JAK-2 protein and ERBB2/HER2 protein, for LC they were CD133, EpCAM, AFP, Protein Plk1 and hnRNP A1 proteins, for pancreatic cancer they were (PAUF), CD71, C/EBP, CSV, CCKBR, HMGA proteins and lastly for CRC it was EpCAM protein. As for applications of aptamers in diagnosis or prognosis and chemotherapy, we also identified the key aptamers in esophageal, gastric, liver, pancreatic, and CRCs. In EC, the aptamers S2, S3, and S8 demonstrated characteristics to be effective diagnosis and prognosis tools. In GC, the aptamers cy-apt-20 and aptNCL demonstrated roles as cancer biomarkers and as drug delivery agents, respectively. In LC, aptamers that contributed as cancer biomarkers and diagnostic or prognostic tools were identified, and they were JHIT1 to JHIT7, Apt-07S, yl19, yl19a, yl23, and LY-1. In pancreatic cancer, M9-5, Aptamer 1 & 146 were identified as diagnostic markers, and the chemotherapeutic drug delivery methods used were through the determined molecular targets such as ALPPL-2, EGFR, and CD71 proteins. As for CRC, there was only one aptamer identified as a diagnostic or prognostic tool, ApC1 and the drug delivery method was through aptamers loaded with Dox, Apt-HJ-Dix, and Apt-NP-DOX. Aptamers-based cancer treatments, diagnosis, or prognosis have and are still facing many obstacles prior to reaching the end user markets due to their inadequate sensitivity and specificity as cancer diagnostic and prognostic tools. Although aptamers have drawbacks, there is still a crucial need for aptamer-associated cancer biomarkers and molecular targets to diagnose, diagnose, and treat GI cancers. Additionally, current studies on the application of aptamers are limited to common GI cancers, such as esophageal, gastric, liver, pancreatic, and colorectal. Thus, it is necessary to also investigate their applicability in the less common GI cancers like anal cancer. Further studies need to be done to determine aptamers with high specificity and affinity to GI cancers for overcoming insufficient sensitivity, effective molecular targets as potential cancer biomarkers, the functionality of relevant targets, and aptamers in GI cancer prognosis, diagnosis, and treatment. Currently, the application of aptamers in pre-clinical phases is limited to several types of GI cancers, and aptamers should be widened out with their potential applications and tested for their function as diagnostic, prognostic, bio-sensing, or imaging tools as well as therapeutic agents, targeting molecular targets, and as effective drug delivery systems.

## Figures and Tables

**Table 1:** Comparison among RNA, DNA, and peptide aptamers.

Properties	RNA Aptamers	DNA Aptamers	Peptide Aptamers
Structure	Limited due to chemical differences (O-H bond)	Limited due to chemical differences (C-H bonds)	Limited due to the presence of scaffold protein
Formation of aptamers	Able to form more secondary-dimensional structures than DNA aptamers	Able to form a limited type of secondary-dimensional structures than RNA aptamers	Able to form limited types of secondary-dimensional structures due to the scaffold protein
Target binding site	Binds to the loop region(s) and undergoes structural conformational changes	Binds to the loop region(s) and undergoes structural conformational changes	Binds to the target with the short peptide sequence, limited due to the sequence present in a scaffold protein

**Table 2:** The diagnostic or prognostic roles of aptamers in GI cancers.

**Table 3:**
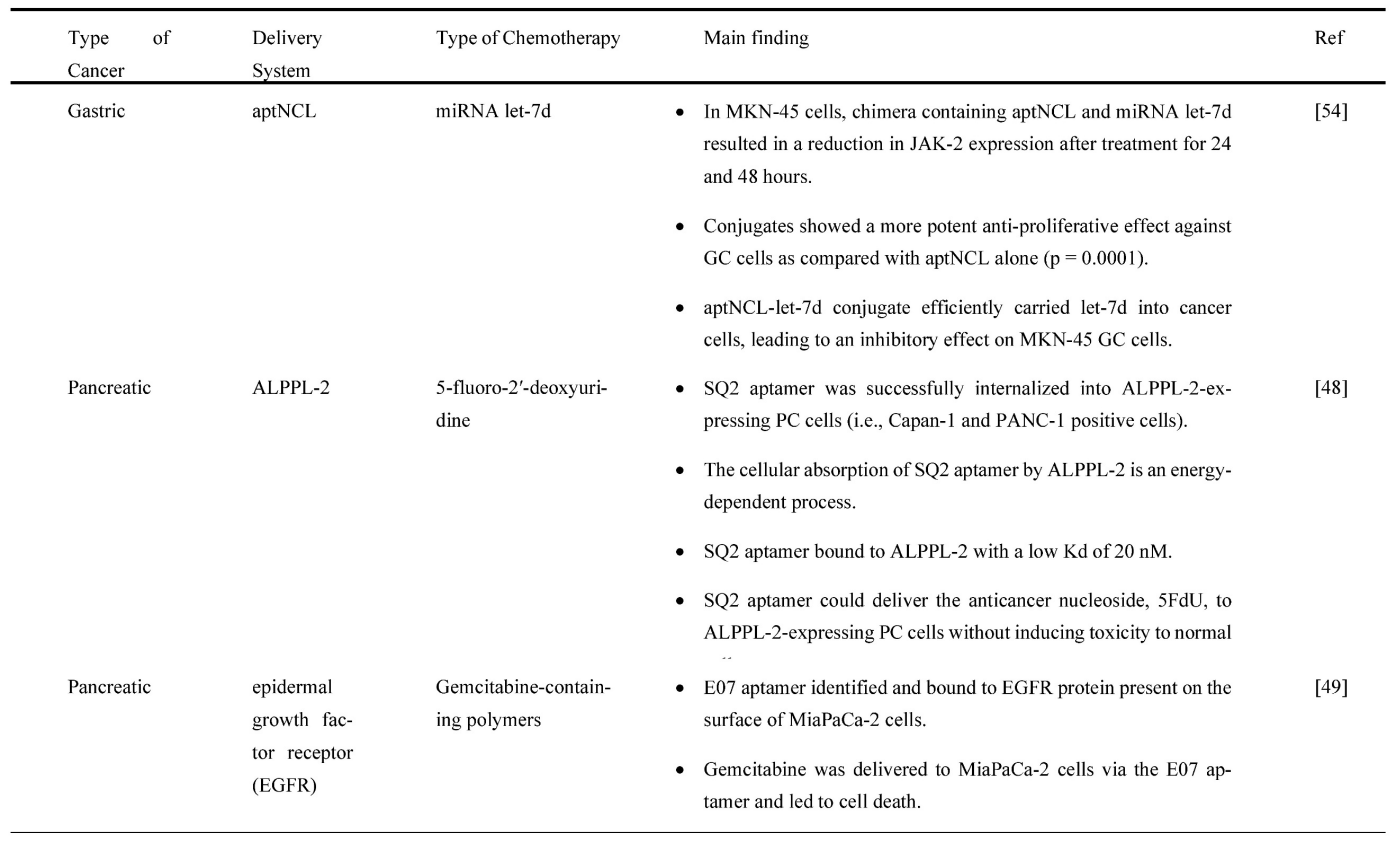

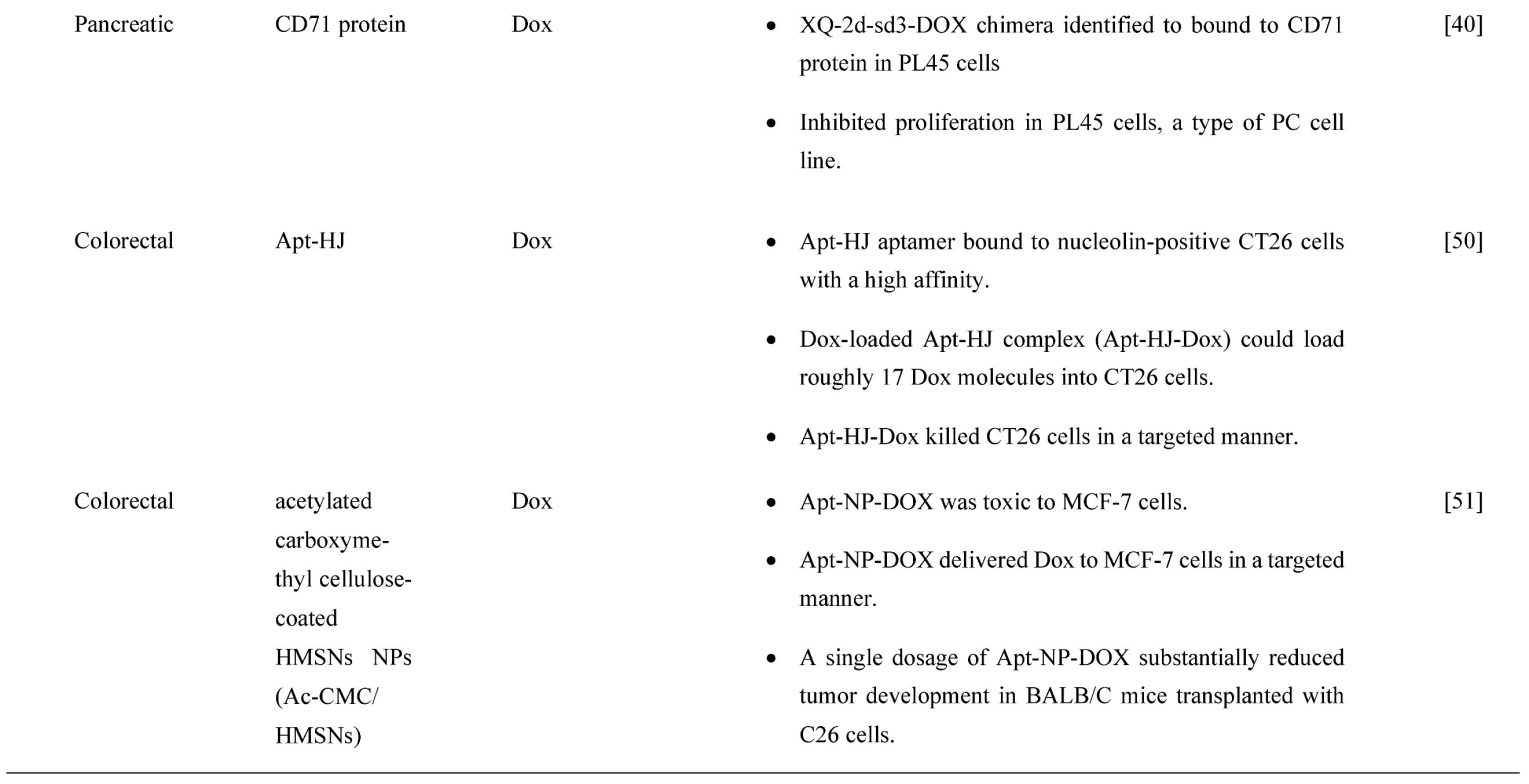
The types of chemotherapy cancer treatment and its drug delivery system for these different types of GI cancers.

**Table 4:**
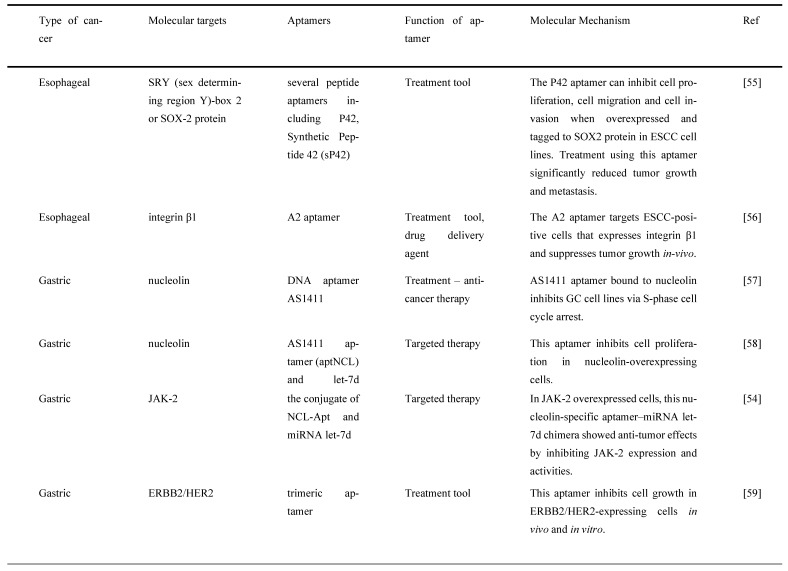

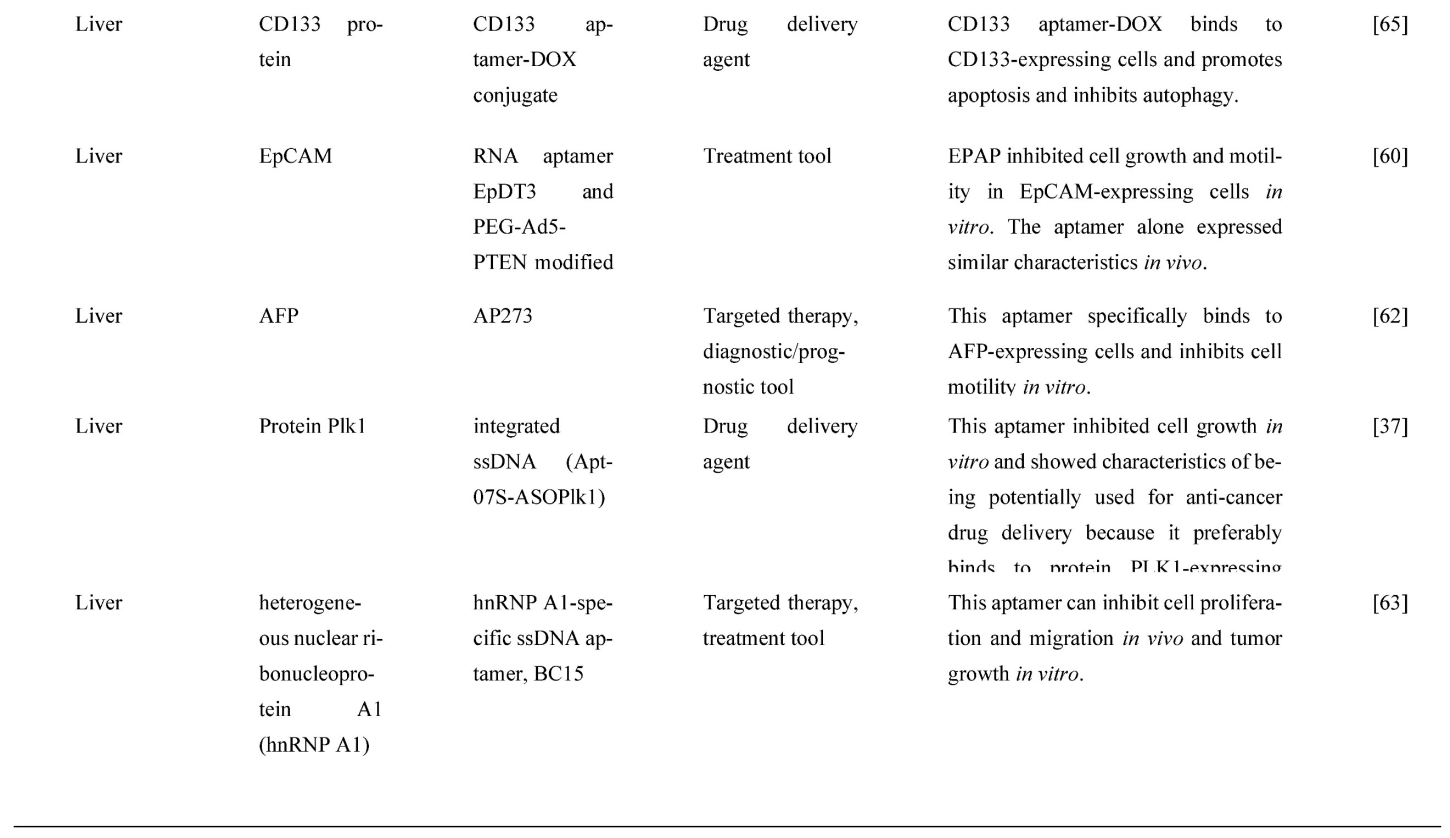

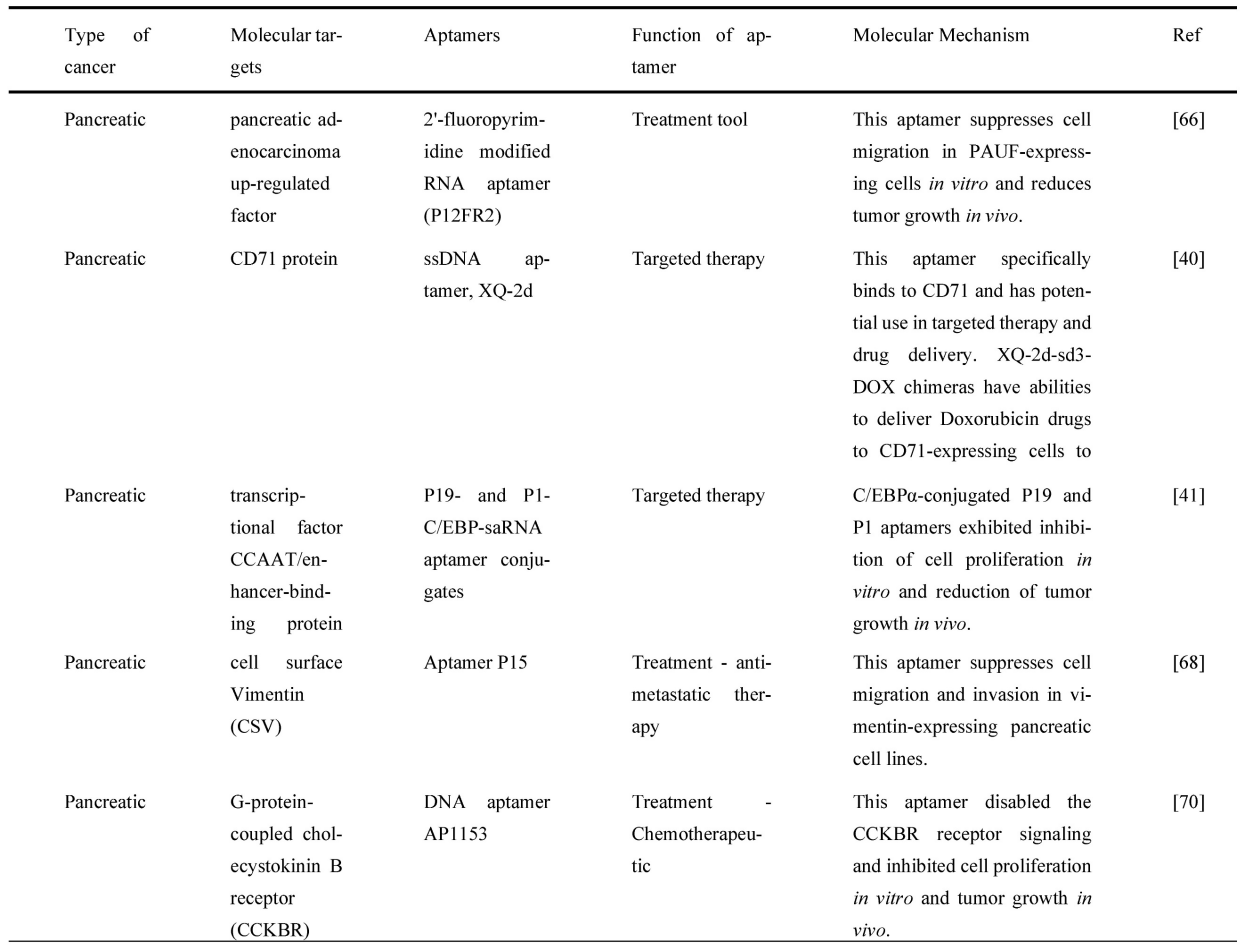

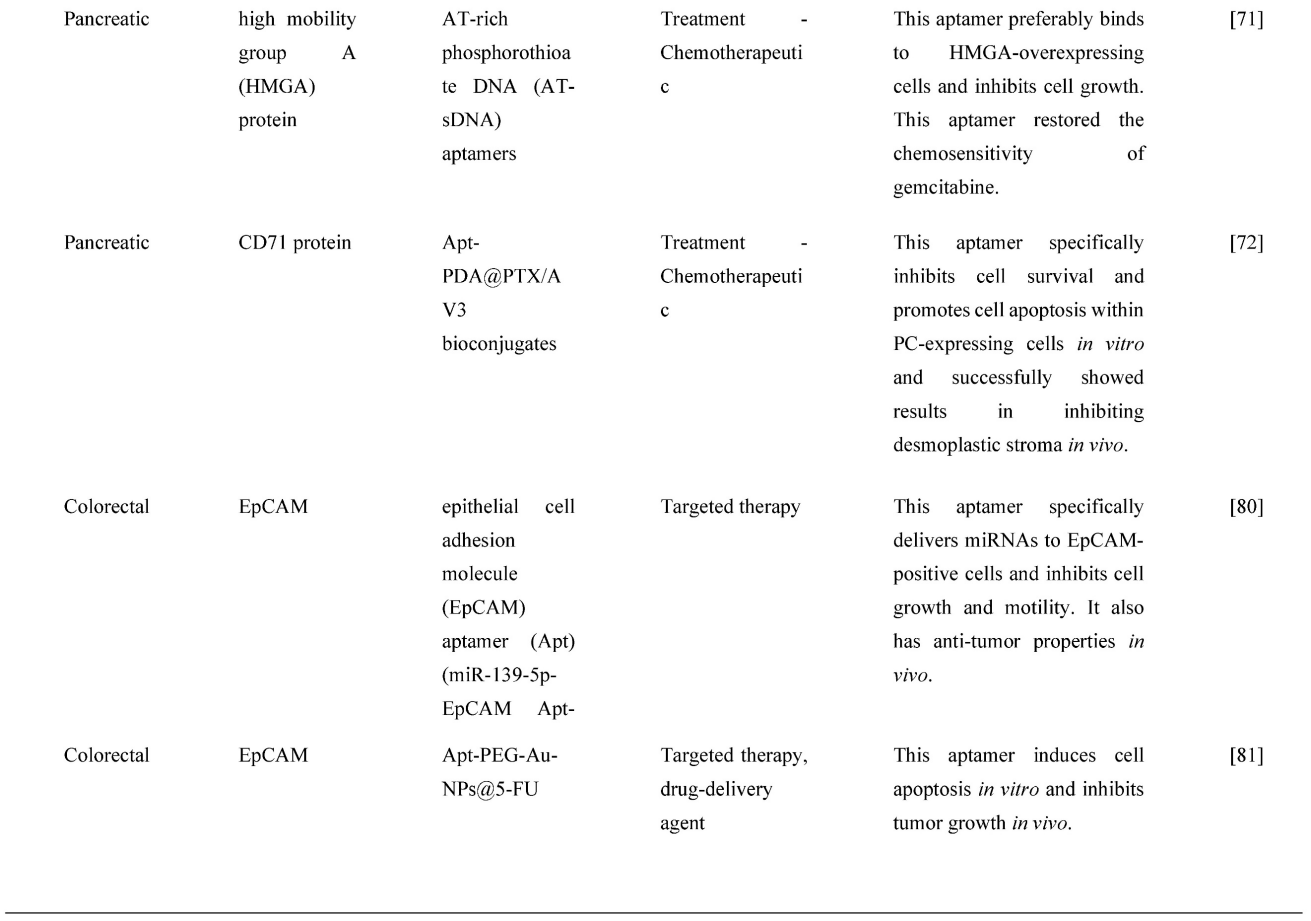
Molecular targets and aptamers for specific type of GI cancers by literature search. The respective functions and molecular mechanisms of aptamers are mentioned below.

## Abbreviations

GI: Gastrointestinal cancer
SELEX: Systematic evolution of ligands by exponential enrichment
CRC: colorectal cancer
GC: stomach/gastric cancer
LC: liver cancer
EC: esophageal cancer
PC: pancreatic cancer
hcG: human chorionic gonadotrophin
CEA: carcinoembryonic antigen
AFP: alpha-fetoprotein
ERBB2/HER2: human epidermal growth factor receptor 2
DNA: deoxyribonucleic acid
RNA: ribonucleic acid
NPs: nanoparticles
PCR: polymerase chain reaction
LOD: limit of detection
ESCC: esophageal squamous cell carcinoma
FITC: fluorescein isothiocyanate
CK19: Cytokeratin 19
GPC3: Glypican-3
HCC: Hepatocellular carcinoma
ssDNA: single-stranded DNA
CD71: transferring receptor-1
Tf: transferrin
SET: surface energy transfer
PDAC: pancreatic ductal adenocarcinoma
MMP14: Matrix Metallopeptidase 14
THY1/CD90: Thy-1 membrane glycoprotein
NUPACK: Nucleic Acid Package
Kd: dissociation constant
SDS-PAGE: sodium dodecyl sulfate-polyacrylamide gel electrophoresis
EpCAM: epithelial cell adhesion molecule
ER: estrogen receptors
PR: progesterone receptors
ASODN: antisense phosphorothioate oligodeoxynucleotide
Plk1: polo-like kinase-1
PBS: Phosphate-buffered saline
CypB: Cyclophilin B
CSC: cancer stem cells
FACS: Fluorescence-activated cell sorting
EphA2: ephrin type-A receptor 2
miRNA: microRNA
rDNA: ribosomal DNA
Tipα: TNF-α inducing protein
H.pylori: Heliobacter pylori
JAK-2: Janus kinase 2
ELISA: enzyme-linked immunosorbent assay
HDF: human derma fibroblast
TAMRA: Tetramethylrhodamine
ALPPL-2: alkaline phosphatase placental-like 2
GPI-AP: glycosylphosphatidylinositol-anchored proteins
5FdU: 5-fluoro-2'deoxyuridine
EGFR: epidermal growth factor receptor
Dox: Doxorubicin
dsDNA: double stranded DNA
CHO: Chinese hamster ovary
IC50: half maximal inhibitory concentration
HMSNs: hollow mesoporous silica NPs
EAC: esophageal adenocarcinoma
SOX2/SRY: sex-determining region Y-box 2
integrin β1: integrin beta 1
STAT: Signal transducer and activator of transcription
HMGA2: high mobility group A2
c-MYC: cellular MYC
mRNA: messenger RNA
hnRNP A1: heterogeneous nuclear ribonucleoprotein A1
siRNPA1: siRNA-specific for hnRNP A1
PAUF: pancreatic adenocarcinoma up-regulated factor
C/EBP: CCAAT/enhancer binding protein
saRNA: small activating RNA
EMT: epithelial mesenchymal transition
MMP3: matrix metalloproteinase-3
CCKBR: cholecystokinin B receptor
HMGA: high mobility group A
AT-sDNA: AT-rich phosphorothioate DNA
PDA: paclitaxel (PTX)-modified polydopamine
5-FU: 5-fluorouracil
S100P: S100 Calcium Binding Protein P
CXCL12/SDF1: chemokine CXCL12
